# Poroptosis: A form of cell death depending on plasma membrane nanopores formation

**DOI:** 10.1016/j.isci.2022.104481

**Published:** 2022-05-30

**Authors:** Hao Li, Zihao Wang, Xiaocui Fang, Wenfeng Zeng, Yanlian Yang, Lingtao Jin, Xiuli Wei, Yan Qin, Chen Wang, Wei Liang

**Affiliations:** 1 Protein & Peptide Pharmaceutical Laboratory, Institute of Biophysics, Chinese Academy of Sciences, Beijing 100101, P. R. China; 2 Key Laboratory for Biomedical Effects of Nanomaterials and Nanosafety, and Key Laboratory of Standardization and Measurement for Nanotechnology, National Center for Nanoscience and Technology, Chinese Academy of Sciences, Beijing 100190, P. R. China; 3 University of the Chinese Academy of Sciences, Beijing, 100049, P. R. China; 4 Department of Molecular Medicine, UT Health San Antonio, San Antonio, TX 78229, USA

**Keywords:** Cell biology, Functional aspects of cell biology, Cancer

## Abstract

Immunogenic cell death (ICD) in malignant cells can decrease tumor burden and activate antitumor immune response to obtain lasting antitumor immunity, leading to the elimination of distant metastases and prevention of recurrence. Here, we reveal that ppM1 peptide is capable of forming irreparable transmembrane pores on tumor cell membrane, leading to ICD which we name poroptosis. Poroptosis is directly dependent on cell membrane nanopores regardless of the upstream signaling of cell death. ppM1-induced poroptosis was characterized by the sustained release of intracellular LDH. This unique feature is distinct from other well-characterized types of acute necrosis induced by freezing-thawing (F/T) and detergents, which leads to the burst release of intracellular LDH. Our results suggested that steady transmembrane-nanopore-mediated subacute cell death played a vital role in subsequent activated immunity that transforms to an antitumor immune microenvironment. Selectively generating poroptosis in cancer cell could be a promise strategy for cancer therapy.

## Highlights

• ppM1 rapidly aggregates on the plasma membrane to form stable irreparable nanopores
• ppM1-formed nanopores induce ICD in tumor cells
• Definition of Subacute cell death
• ppM1 synergizes PD-1 antibody and therapeutic vaccine in murine tumor models

## Graphical abstract

**Figure:**
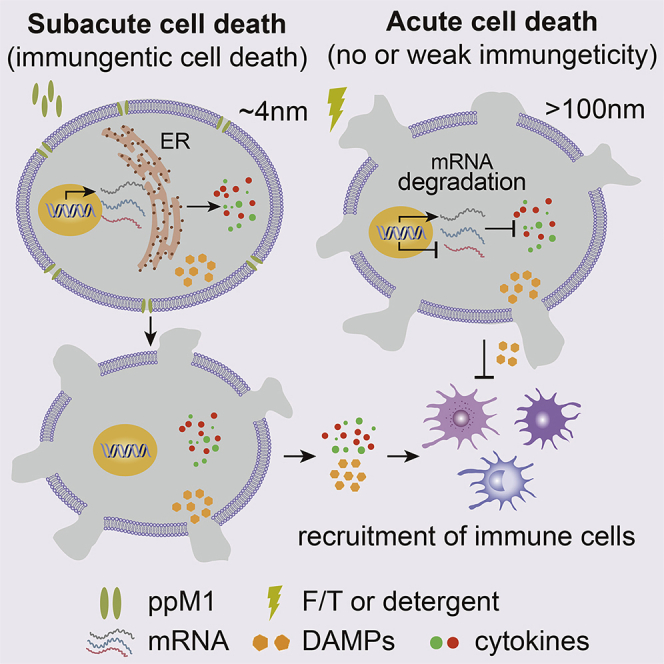

## Introduction

Immunogenic cell death (ICD) represents a unique form of cell death that is capable of provoking an adaptive immune response against dead-cell-associated antigens for immunocompetent hosts (Galluzzi et al., 2018; Pol et al., 2015). It has been widely recognized that optimal treatments for cancer should not only kill malignant cells but also provoke patient’s own antitumor immune response. Therefore, inducing malignant cells' immunogenicity death could provide an important means of cancer therapy. The crux of treatment is immunogenicity of dying cells, which relies on a combination of antigenicity and adjuvanticity. Mutated antigens exposed from dead malignant cells can initiate an adaptive immune response when these dying cells emit adjuvant signals during the processes of cellular stress and death. As the consequence of cellular stress and death, DAMPs (damage-associated molecular patterns), such as ATP, HMGB1, can operate as natural adjuvants to activate PRRs (pattern recognition receptors) signaling to provide the ideal precondition for the initiation of antigen-specific immune responses(Broz and Monack, 2013; Cao, 2016; Fuchs and Steller, 2015; Galluzzi et al., 2017; Yatim et al., 2017). Under the selection pressure of immune system, however, both pathogens and malignant cells have evolved limited adjuvanticity and therefore can escape from immune surveillance. During the oncogenesis, malignant cells prefer adopting a pattern of death with no DAMPs released, such as apoptosis, to limit adjuvanticity to escape from immune surveillance. Thus, releasing DAMPs from dying malignant cells is crucial for eliciting a robust antitumor immunity.

Multiple cell death forms have been reported; however, their roles engaging in the immune response are still poorly understood. It has been suggested that apoptosis is implicated in immunotolerance, while necrosis is associated with inflammation and immunogenicity The mechanistic basis for the differential immunogenicity between apoptosis and necrosis may stem from their different ability to maintain integrity of membrane and release DAMPs (Nagata and Tanaka, 2017). Distinct membrane morphological hallmarks between apoptosis and necrosis: apoptotic cells membrane repeats the process of blebbing and retraction to form apoptotic bodies, which packed with cellular contents to avoid releasing DAMPs, while necrosis is manifested as membrane swell and rupture, cellular contents extravasation, and releases a variety of intracellular DAMPs into the extracellular environment to trigger severe local inflammation (Elmore, 2007; Messmer et al., 2019; Zhang et al., 2018). Those observations imply that the membrane integrity of dying cells may dictate the nature of the follow-up immune response: immunotolerance or immunogenicity. Through careful analysis of membrane damage during necrosis, such membrane rupture is primarily derived from the membrane pores formed by relevant executional protein (Liu and Lieberman, 2020; Zhang et al., 2018). More specifically, necrosis comprises necroptosis and pyroptosis, the former of which is executed by mixed lineage kinase-like (MLKL). MLKL can form octamer complexes and translocate to and span across the plasma membrane to form membrane pores after phosphorylation by upstream signals of necroptosis (Chen et al., 2014; Su et al., 2014; Wang et al., 2014; Weinlich et al., 2017). Pyroptosis critically depends on the formation of plasma membrane pores by members of the gasdermin protein family, which can multimerize and insert into the target membrane to form large nanopores in the shape of β barrels (Ding et al., 2016; Liu et al., 2016; Ruan et al., 2018). Both necroptosis and pyroptosis can release multiple DAMPs and pro-inflammatory cytokines and give rise to inflammation and immunogenicity (Kaczmarek et al., 2013; Messmer et al., 2019; Nagata and Tanaka, 2017; Weinlich et al., 2017). These observations indicate that there is certain undefined relationship between the loss of membrane integrity by membrane pore and immunogenicity of cell death. In addition, two previous reports demonstrated that membrane pores generated by voltage or protein can mediate antitumor immune response: one executed irreversible electroporation on membrane giving rise to release DAMPs and reversed resistance to immune checkpoint blockade in melanoma and pancreatic cancer model; the other reported that applying purified GSDMA3 to form large pores on membrane resulted in immunogenic cell death, independent of upstream signal of pyroptosis, and stimulated the antitumor immunity in 4T1 model (Wang et al., 2020; Zhao et al., 2019).

Based on previous observations and new findings in this study, we attempt to propose a new form of cell death, poroptosis, which directly depends on cell membrane nanopores regardless of the upstream signaling of cell death. Poroptosis of malignant cells is sufficient to independently cause immunogenic cell death and trigger antitumor immune response. It has been reported that peptides whose amino acid sequence derived from p53 protein, could permeate cell membrane and even form membrane pores on tumor cells (Kanovsky et al., 2001; Rosal et al., 2005; Sarafraz-Yazdi et al., 2015). We have identified a peptide (pM1) derived from the fragments of p53-MDM2 binding domain of p53 protein that directly forms irreparable nanopores on the cell membrane of tumor cells, leading to intracellular LDH sustained release, and ultimately, ICD. The pM1-induced cell death was characterized by the sustained release of intracellular LDH, which is distinct from other well-characterized types of cell death induced by FT and detergents that leads to the burst release of intracellular LDH. Our results suggest that the manipulation of poroptosis may be exploited to controllably destroy tumor cells and to modulate immune responses.

## Results

### pM1 and ppM1 require plasma membranes disruption to induce cell death in tumor cells

**Figure 1:**
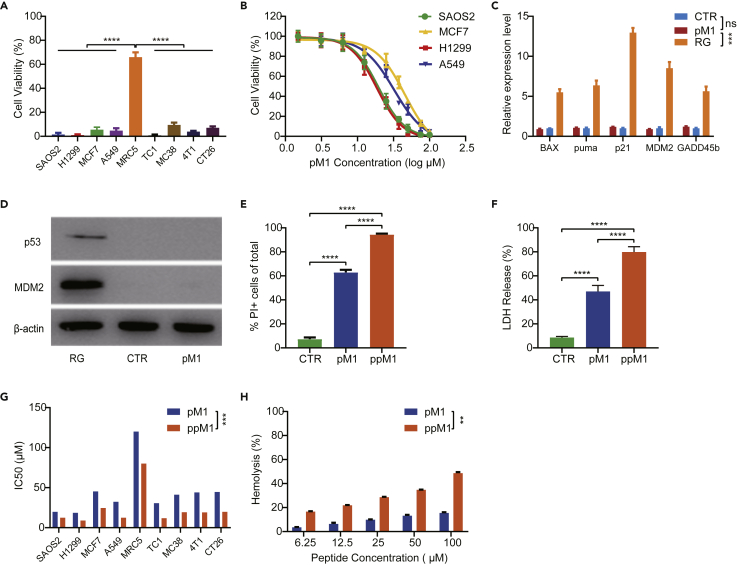
pM1 and ppM1 induced tumor cell death by disrupting plasma membranes (A) Cytotoxicity evaluation by MTT experiment on several tumor cell lines treated with 100 μM pM1 for 24 h. (B) Survivorship curves of p53-wild (MCF7 and A549) and p53-null (SAOS2 and H1299) cell lines treated with pM1 for 24 h. (C and D) Relative expression of p53 and its downstream genes on A549 cells (Bax, puma, p21, MDM2, and GADD45b) after pM1 treated for 24 h, detected by Q-PCR (C) and Western Blot (D). (E) Proportion of propidium iodide positive (PI+) cells measured by flow cytometry after co-incubating MC38 cells with 30 μM pM1 or ppM1 for 30 min. (F) Detection of LDH release after treating MC38 cells with 50 μM pM1 or pM1 for 6 h. (G) Comparison of IC_50_ between pM1 and ppM1, measured by MTT experiments. (H) Hemolysis comparison between pM1 and ppM1. Representative of 3 independent experiments in (A–C) and (E–H), the error bars represent SDs. (C) was analyzed with 1-way ANOVA. (G) and (H) was analyzed with two-tailed paired t test. Other data were analyzed with two-tailed unpaired t test. ∗∗p < 0.01; ∗∗∗p < 0.001; ∗∗∗∗p < 0.0001. (See also Figure S1–S4). Pubmed Partial Author articletitle stitle stitle Volume PAGE.

We designed and screened one peptide, named pM1; it exhibited a broad-spectrum antitumor activity (Figures S1A and S1B). pM1 derived from the p53-MDM2 binding domain of p53 protein consists of 29 amino acids and possesses the typical characteristics of amphipathic structure and multiple net positive charges, which partly determine its membrane-permeation potential. We first evaluated the antitumor efficacy of pM1 at a high concentration (100 μM) on various tumor cell lines *in vitro*. As shown in Figure 1A, pM1 was cytotoxic to various cancer cell lines, causing almost 100% cell death at that concentration on both human cancer cell lines (SAOS2, H1299, MCF7, and A549) and mouse cancer cell lines (TC1, MC38, 4T1, and CT26), but did only mildly affect normal human cells (MRC5). This could be explained by the fact that cancer cell membranes often overexpressed negatively charged macromolecules, such as phosphatidyl serine and proteoglycans (Dube and Bertozzi, 2005; Eksteen et al., 2017; Utsugi et al., 1991). The increasing of anionic character in cancer cells could make them be vulnerable to the positively charged pM1 than noncancerous cells. By utilizing amino acid substitution strategies (Figure S2A), we confirmed that the positively charged arginine in pM1 plays a critical role in its tumoricidal function. In addition, we also found that the hydrophobicity provided by the non-polar amino acid leucine in pM1 also played an important role in its tumoricidal function (Figure S2B). p53 protein is considered as an extremely important tumor suppressor and mainly as a transcription factor for regulating the cell cycle and apoptosis. We first considered to verify that whether the tumoricidal efficacy of pM1 had a dependency on p53 signal pathway. From Figure 1A, we also observed that pM1 caused both p53-wild (MCF7 and A549) and p53-null (H1299 and SAOS2) cell death, suggesting a cytotoxic mechanism independent of p53. To test this speculation, we compared the survivorship curve of both the p53-wild (A549, MCF7) and the p53-null (H1299, SAOS2) tumor cell lines (Muller and Vousden, 2014; Tovar et al., 2006; Wachter et al., 2017), at a concentration gradient of pM1 and detected the downstream signaling of p53 following pM1 treatment by Western Blot and Q-PCR. The results showed that pM1 possessed nearly an equal cytotoxicity to both the p53-wild and the p53-null cell lines (Figure 1B). In addition, A549 cells following pM1 treatment had no difference to the accumulation of p53 and MDM2 protein (Figure 1D), as well as the transcriptional activation of the downstream signals of *p53* pathway, including *Bax*, *puma*, *p21*, *MDM2*, and *GADD45b* (Figure 1C), compared to RG, an inhibitor that blocks the interaction between p53 and MDM2, as a positive control (Tovar et al., 2013). On the other hand, pM1 rapidly facilitated the entry of nucleic acids dye propidium iodide (PI) into cells within 30 min (Figure 1E) and the accumulated release of lactate dehydrogenase (LDH) for 6 h (Figure 1F), indicating that pM1 induced cell membrane damage and resulted in cell necrosis. Taken together, these results demonstrated that pM1-induced necrosis of cancer cells in this study was due to direct disruption of cell membrane rather than depending on p53 signal pathway.

It is a common problem that the internalization of peptides into cytoplasm severely limits the ability of retention in cell membrane. Thus, decreasing the amount of internalized pM1 can improve its ability to damage membrane integrity as well as enhance its antitumor efficacy. Therefore, we conjugated a palmitic acid with pM1 at its amino terminus, renamed as it as ppM1. The hydrocarbon chains of decorated palmitic acid could insert into outer leaflet of the phospholipid bilayer (Colsky and Peacock, 1989; Iwanaga et al., 2009).The palmitic-decorated peptides could tightly anchor to cell membranes, and consequently delay their internalization. Next, we examined the capability of pM1 and ppM1 in destroying cell membrane by detecting the amount of PI permeabilized and LDH released in MC38 cells. Both pM1 and ppM1 significantly enhanced the entry of PI into the cells. At the same concentration (30 μM), more than 90% of the cells were PI positive after ppM1 treatment while only about 60% of the cells stained by PI after pM1 treatment (Figure 1E). Meanwhile, ppM1 treatment led to much more release of LDH than that of pM1 treatment (Figure 1F). These results suggested that palmitic acid conjugated to pM1 enhanced the loss of cell membrane integrity as well as the ability to induce cell necrosis. Correspondingly, the inhibitory concentrations (IC_50s_) of ppM1 to all experimental cancer cell lines tested were significantly lower than that of pM1 (Figure 1G). In addition, conjugated with a palmitic acid, the hemolytic capacity of pM1 was improved to some extent (Figure 1H), further suggesting ppM1 could have a stronger potential to destroy plasma membrane than pM1. Moreover, similar to pM1, ppM1 was cytotoxic to various cancer cell lines, causing almost 100% cell death at a certain concentration of 100 μM on both human (SAOS2, H1299, MCF7, and A549) and mouse (TC1, MC38, 4T1, and CT26) cancer cell lines, but imposed only mild effects on human embryonal lung fibroblasts (MRC5), murine embryonal fibroblasts (NIH/3T3), and primary fibroblasts from mouse tail tissues (Figure S3). Although ppM1 has the improved hemolysis, systemic toxicity was not seen after the mice received intratumor injection with 25 mg/kg of ppM1 (Figures S4C and S4D). Furthermore, we also compared the oncotherapy outcomes of pM1 and ppM1 *in vivo* in MC38 tumor-bearing C57BL/6 mice or H1299 tumor-bearing nude mice. Mice were injected intratumorally with 25 mg/kg of pM1 or ppM1, respectively, and both pM1- and ppM1-treated mice showed significantly lower average tumor volumes than the untreated mice. Particularly, ppM1 exhibited much better therapeutic efficacy than pM1 both in MC38 model (Figure S4A) and in H1299 model (Figure S4B). Taken together, these results demonstrated that both pM1 and ppM1 are capable of causing the loss of membrane integrity, which in turn leads to tumor cell death.

### Nanopore formation requires ppM1 rapid aggregation on plasma membrane

**Figure 2:**
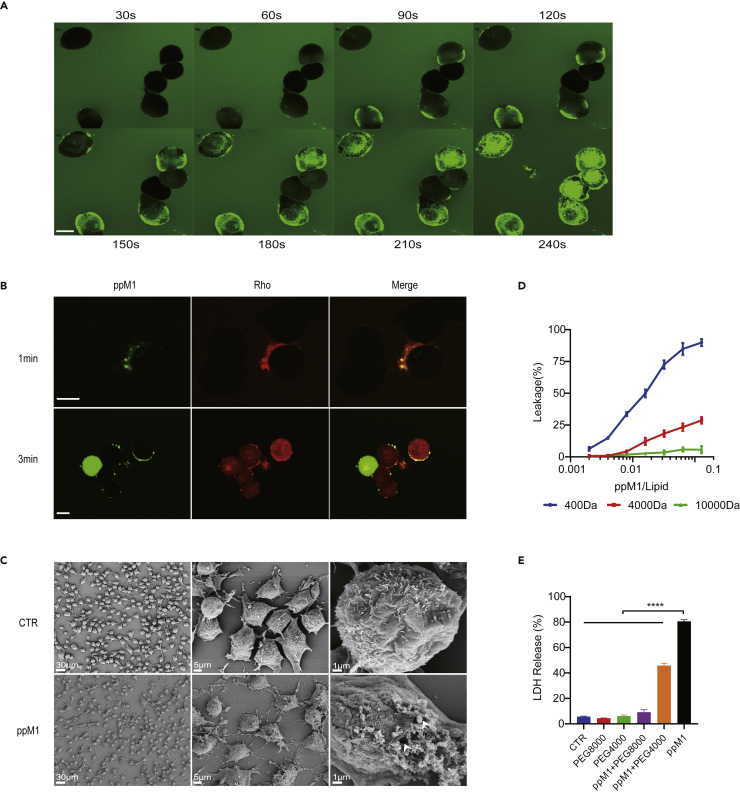
ppM1 rapidly aggregated on plasma membrane and formed nanopore (A) Confocal images of the treated MC38 cells and fluorescein isothiocyanate (FITC)-labeled ppM1 (10 μM) were added to the cell medium and quickly observed at different time points. Scale bars, 10 μM. (B) Confocal images of the treated MC38 cells, FITC-labeled ppM1 (10 μM), and rhodamine (100 ng/mL) were added to the cell medium and quickly observed at serial time nodes. Scale bars, 10 μM. (C) SEM images of the MC38 cells were treated with or without ppM1 (10 μM) for 10 min. Scale bars, left 30 μM, middle 5 μM, right 1 μM. The white arrows indicated membrane “pore” or “hole”. (D) Liposome leakage after ppM1 treatment was monitored by measuring the encapsulated fluorescence relative to that of Triton X-100 treatment. (E) LDH release from MC38 cells after 30 μM ppM1 treatment for 6 h with or without PEGs (4000 or 8000). Representative of 3 independent experiments in (D–E), and error bars represent SDs. (E) were analyzed with 1-way ANOVA. ∗∗∗∗p < 0.0001. (See also Figure S5).

Next, we sought to investigate how pM1 or ppM1 permeates and disrupts plasma membranes and whether it is through the formation of steady transmembrane pores. We dynamically traced the location of FITC-labeled ppM1 (F-ppM1) on MC38 cells within a few minutes by laser confocal microscope, as shown in Figure 2A. Once F-ppM1 added to the medium, F-ppM1 rapidly accessed to membrane and agglomerated into larger speckle particles in 1 min. And with the increase of incubating time, more F-ppM1 were observed to accumulate in membrane and then permeated into the inside of the cells (Figure 2A). A few minutes later, fluorescence dispersed throughout the whole cell, especially the green fluorescence intensity located in the plasma membranes and the nucleus was higher than the other places (Figure 2A). The cells were treated for 2 h, FITC-labeled pM1 was mainly located in the nucleus, and the integrated plasma membrane structure could not be seen (Figure S5A). The results suggested that ppM1 preferentially bound to plasma membrane at first and crossed membrane to condense in the nucleus, which could diminish the damage to the subcellular organelle membrane. Indeed, we observed the vitalized mitochondria after 2 h treatment with 10 μM of pM1 (Figure S5B). To further investigate whether ppM1 can form transmembrane pores, we added carboxy-tetramethyl-rhodamine into the medium, a poorer cell membrane permeability fluorescent dye (Marinova et al., 2005). Only at very early timepoint after F-ppM1 addition, the loci of F-ppM1-anchored membranes (where pp-M1 aggregation formed) could co-localize with the rhodamine fluorescence, while on the contrary, rhodamine fluorescence did not co-localize with F-ppM1 if pp-M1 did not aggregate (Figure 2B top). Later, F-ppM1 and rhodamine were co-localized on the cell membranes, and then rhodamine molecules gradually dispersed into the whole cell (Figure 2B bottom), suggesting the possibility of ppM1-mediated formation of plasma membrane pores. These membrane pores were clearly observed by scanning electron microscope, as shown in Figure 2C. The cells treated with 10 μM of ppM1 for 10 min showed the unintegrated plasma membrane structure and the pore- or the hole-like structures on membranes.

Next, we desired to experimentally determine the size of ppM1-formed membrane pores. We adopted liposomes to simulate cell membranes and determined the size of membrane pores by the leakage of fluorescein with increasing molecular weight from liposomes following ppM1 treatment. The result showed that carboxy-fluorescein (MW, ∼400 Da) as well as FD4 (FITC-dextran, ∼4000 Da) leaked out of liposomes through pores formed by ppM1, and the amount of leakage increased gradually with the increasing concentrations of ppM1 (Figure 2D). However, the leakage of FD10 (FITC-dextran, ∼10,000 Da) from liposome was hardly seen with ppM1 treatment at different concentrations. Also, we directly determined that by means of adding different molecular weight osmotically active agents to cell media (Pedrera et al., 2021; Ros et al., 2017). We assessed the effect of PEGs of different sizes on the extent of LDH release, the hallmark of plasma membrane rupture, and cell necrosis. As shown in Figure 2E, LDH release resulted from ppM1 was compromised by adding PEG4000. Furthermore, it was completely counteracted in the presence of PEG8000. Moreover, removal of PEG8000 restored ppM1-induced MC38 cell death, which further proving that the membrane pores are stable (Figure S6). Taken together, these data allow us to estimate the approximate size of ppM1-formed membrane pores to be roughly between 4 and 8 kDa.

The ppM1-formed membrane pore size is larger than antimicrobial peptides (AMPs)-formed membrane pore size, which only permits the smallest dyes encapsulated, with molecular weights of ∼400 Da, to leak out of the liposome (Wang et al., 2016), This may explain the weak cytotoxicity of AMPs on eukaryotic cells as well as the stronger antitumor efficacy of ppM1. At the same time, the size of ppM1-formed membrane pore was similar with that formed by MLKL. MLKL induces necroptotic pores and is bigger in size than PI but smaller than FD10 dextran and about 4 nm in diameter, but was obviously smaller than gasdermin-formed membrane pore size, which could release dextran with molecular masses of 3 or 10 kD but not 40 kD from the liposome (Ding et al., 2016; Ros et al., 2017; Wang et al., 2020). Taken together, these results demonstrated that ppM1 can rapidly aggregate on plasma membrane and form about 4 nm of nanopores, and these membrane pores can induce ICD on tumor cells and activate antitumor immune response, as described below.

### ppM1 treatment induces ICD in tumor cells

**Figure 3:**
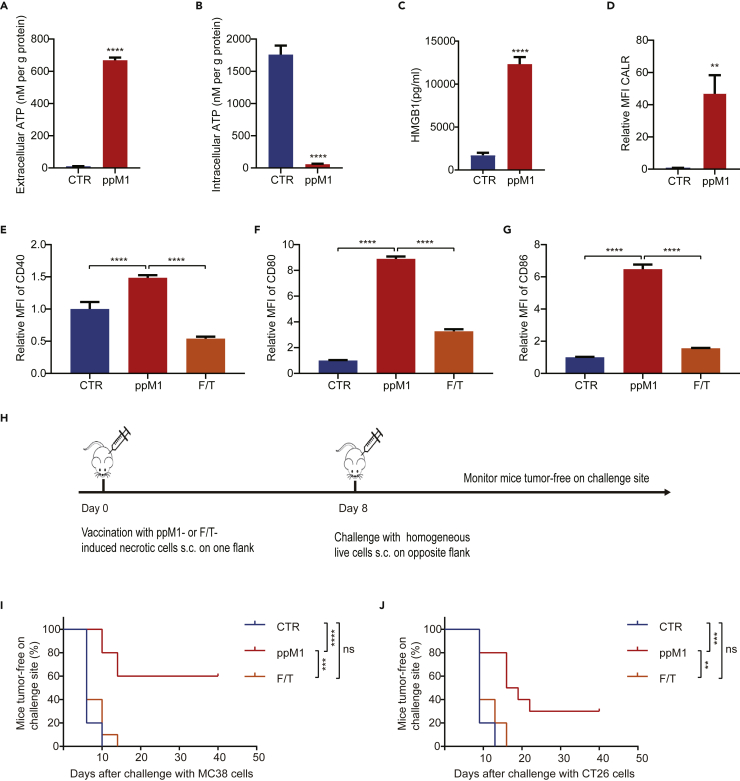
ppM1 treatment induced ICD of tumor cells both *in vitro* and *in vivo* (A–D) Extracellular ATP (A) and intracellular ATP (B) detection after ppM1 treatment for 2 h, extracellular HMGB1 (C) detection after ppM1 treatment for 8 h, exposure of calreticulin (D) on membrane after ppM1 treatment for 1 h. (E–G) Flow cytometry measurements of BMDC maturation markers (CD40, CD80, and CD86) after coculturing with necroptotic MC38 cells induced respectively by ppM1 or freeze-thawing cycles. (H) The schedule of prophylactic tumor vaccination experiments in Figures 3I and 3J. (I and J) Rechallenges of tumor inoculation after immunization with 3×10^6^ necroptotic cells induced by ppM1 or necrotic cells induced by F/T on both MC38-bearing C57BL/6 (I) (n = 10) and CT26-bearing BALB/c mice (J) (n = 10). Representative of 3 independent experiments in (A–G). All error bars represent SDs. (A–D) was analyzed with two-tailed unpaired t test, (E–G) was analyzed with 1-way ANOVA, (I–J) was analyzed with log rank (Mantel–Cox) test. ∗∗p < 0.01; ∗∗∗p < 0.001; ∗∗∗∗p < 0.0001. (See also Figure S7).

As mentioned above, DAMPs are of vital importance to initiate antigen-specific immune responses, and several of them have been identified as the biochemical correlates of ICD, including the exposure of calreticulin (CALR) on the surface of dying cells, the release of ATP, and high-mobility group box 1 (HMGB1) into the extracellular milieu (Galluzzi et al., 2017). We next investigated whether ppM1 would be able to stimulate the release of DAMPs. After treating with ppM1, the concentration of extracellular ATP (Figure 3A), HMGB1 (Figure 3C), and the amount of exposure of CALR on plasma membrane (Figure 3D) were all dramatically increased, compared to the untreated cells. Correspondingly, the concentration of intracellular ATP (Figure 3B) was decreased dramatically.

Next, we tested the immunogenic properties of ppM1-killed tumor cells *in vitro* by detecting the maturation status of bone-marrow-derived murine dendritic cells (BMDCs) after co-culturing with necrotic cells induced by ppM1 or freezing-thawing (F/T represents one extremely physiochemical stress that mediates membrane crack to induce accidental cell death) overnight. We found that the ppM1-treated cells significantly stimulated the expression of co-stimulatory molecules CD40, CD80, and CD86 (Figures 3E–3G), indicating the maturation of BMDCs. Whereas F/T-treated cells had extremely weak effects on the maturation of BMDCs, when compared with the untreated BMDCs (Figures 3E–3G).

For *in vivo* experiments, we used a well-established prophylactic tumor vaccination model in immunocompetent C57BL/6 and BALB/c mice (Figure 3H) to test the ability of ppM1-induced necrotic tumor cells in activating the adaptive immune system, which is also the generally accepted gold-standard approach to test the ability of a specific agent to induce bona fide ICD (Aaes et al., 2016; Galluzzi et al., 2017; Obeid et al., 2007). Immunization of the mice with ppM1-induced necrotic cells prevented tumor growth on challenge site on both MC38-C57BL/6 model (Figure 3I) and CT26-BALB/c model (Figure 3J), compared with the control mice or with the mice vaccinated with F/T-induced accidental necrotic cells. Together, these *in vitro* and *in vivo* results indicated that ppM1 treatment can generate robust ICD of tumor cells.

### Nanopores on cell membrane formed by ppM1 mediated subacute cell death

Necrosis has long been described as a consequence of extreme physiochemical stress, such as osmotic shock. F/T that can cause membrane crack and release DAMPs, is therefore classified as uncontrolled or accidental cell death (Kaczmarek et al., 2013). Notably, BMDCs co-cultured with the F/T-induced necrotic cells apparently did not alter the maturation status of BMDCs (Figures 3E–3G) and the mice vaccinated with F/T-induced necrotic cells also did not remain tumor-free at the challenge site on both MC38-C57BL/6 and CT26-BALB/c model (Figures 3I and 3J), which are in line with the previously published papers (Aaes et al., 2016; Casares et al., 2005), suggesting that this form of necrotic tumor cells apparently is a lack of or a weak immunogenicity and fails to induce tumor cell into ICD. By contrast, ppM1 disrupted plasma membranes by forming nanopores, and induced significant ICD. What was the difference between these two types of membrane-damages that determine the entirely different immunogenicity on necrotic tumor cell death?

**Figure 4:**
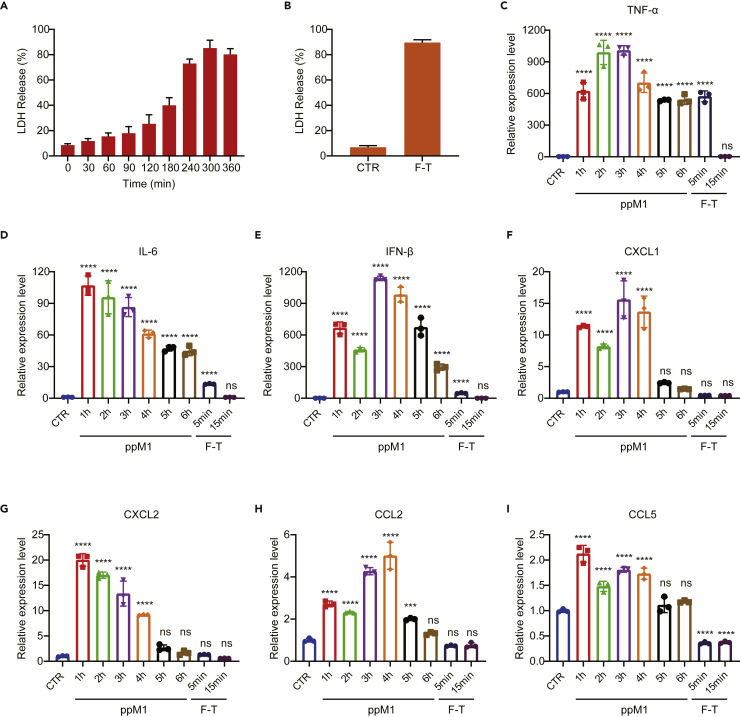
Nanopores on cell membrane formed by ppM1 mediated subacute cell death (A and B) Sustained release of LDH from MC38 cells under 50 μM ppM1 treatment (A) or subjected to freeze-thawing (B). (C–I) Relative expression level of cytokine and chemokine genes following MC38 cells treated by 50 μM ppM1 for indicated time (1, 2, 3, 4, five, or 6 h) or subjected to freeze-thawing once (thawing at 37°C for 5 min or 15 min), *TNF-α* (C), *IL-6* (D), *IFN-β* (E), *CXCL1* (F), *CXCL2* (G), *CCL2* (H), and *CCL5* (I). Data are representative of three independent experiments; all error bars represent SDs. (C–I) was analyzed with 1-way ANOVA. ∗∗p < 0.01; ∗∗∗p < 0.001; ∗∗∗∗p < 0.0001. (See also Figure S7).

Historically, PI and LDH are representatives of two principally categories to evaluate necrotic cell death by determining damage of the plasma membrane. PI is membrane-impermeable dye and does not traverse intact plasma membrane unless existing over 1.5 nm membrane pores. LDH was 135–140 kD intracellular molecules, with the size of about 135 nm in diameter, would be leaked out through impaired plasma membrane from necrotic cells (Bowman et al., 2010; Chan et al., 2013; Imagawa et al., 2016; Jafary et al., 2019; Nesin et al., 2011). Thus, we adopted PI and LDH, two different indicators, to reflect the severity of membrane damage. As shown in Figure 2, ppM1 quickly aggregated on plasma membrane and formed nanopores within a few minutes, correspondingly, nearly all cells were transformed into PI positive within 30 min after co-incubation with ppM1 (Figure 1E), indicating that ppM1 rapidly induced plasma membrane damage. However, the event of LDH leakage was not synchronized with the rapid membrane damage, less than 20% of LDH was leaked out at early 1 h (Figure 4A), indicating that the early nanopores formed by ppM1 was not significantly enough to LDH leak out of the cells. And the accumulation of extracellular LDH increased moderately with prolonging the treated time and reached the maximum around the fifth hour (Figure 4A), suggesting that the ppM1-formed nanopores was relative steady and irreversible beyond the range of cells self-repair, which gradually generate the oversize membrane “pores” or “holes” that lead to cellular macromolecular proteins to leak out of the cells. By contrast, necrosis induced by extreme physiochemical stress, such as F/T or detergents, caused the burst of LDH release from the cells. We observed that nearly or even more than 90% of LDH was released at the first cycle of F/T (Figure 4B), or at the first 30 min after treating with detergents, Triton-X or SDS (Figures S4A and S4B), and the amount of extracellular LDH was not further significantly increased when prolonging incubation time of detergents or increasing the cycle number of F/T. As expected, like F/T, necrotic cells induced by detergents also showed weak or lacking immunogenicity (Figures S4C–S4E).Whereafter, we estimated the expression of cytokines and chemokines during the process of different membrane damage, which were involved in the process of immune activation, such as *TNF-α*, *IL-6*, *IFN-β*, *CXCL1*, *CXCL2*, *CCL2*, and *CCL5*. The gene transcriptional levels of inflammatory cytokines, including *TNF-α*, *IL-6*, and *IFN-β*, were dramatically increased throughout 6 h after ppM1 treatment (Figures 4C–4E). In comparison, although the significant increase in genes expression was also observed after the cell was frozen and thawed at 37°C for 5 min, while 15 min later these genes expression quickly went down to the level of the untreated cells (Figures 4C–4E). For the detergents-treated cells, a comparable genes expression with the untreated cells was observed (Figures S4F–S4H). In addition, the genes transcriptional levels of chemokines, including *CXCL1*, *CXCl2*, *CCL2*, and *CCL5*, were also significant increased throughout at least 4 h after ppM1 treatment (Figures 4F–4I), while there were not significantly increased when the cells were treated by F/T (Figures 4F–4I) or by detergents (Figures S4I–S4L), and among of them, *CXCL1* and *CCL5* were significantly lower than the untreated cells, this could be highly possibly ascribed to the quick mRNAs degradation upon cell membrane broken.

ppM1 preferentially bound to plasma membrane at first, and then crossed the membrane to mainly concentrate in the nucleus, indicating that ppM1 has less damage to subcellular organelles membrane, thus retaining their functions normally during a certain period to proceed the translation of genes. Similarly, a previous study has reported that cytokine mRNA continues to be translated after the formation of MLKL pores (Orozco et al., 2019). In this process, the cells undergo from the initial formation of membrane nanopores to the gradual release of LDH and to the persistently high expression of cytokines, giving a name such a process as subacute cell death (SCD). The main characteristics of SCD are: (1) relative integrated subcellular organelle for a period to continue some physiological activities, such as cytokines transcription and translation, (2) tardiness of membrane disruption, manifested as the sustaining release of LDH.

ppM1-induced SCD by forming membrane nanopores is obviously distinct from the extremely physiochemical stresses-induced accidental necrosis such as F/T and detergents. The former is manifested as forming membrane nanopores and causes SCD with cytokine production, while the latter is manifested as a rapid broken membrane and leads to necrosis with a lack of cytokine production due to quickly degraded cytokine mRNAs. Hence, SCD might be a vital process for generating ICD.

### ppM1 treatment enhanced T cells infiltration and remolded the tumor immune microenvironment

**Figure 5:**
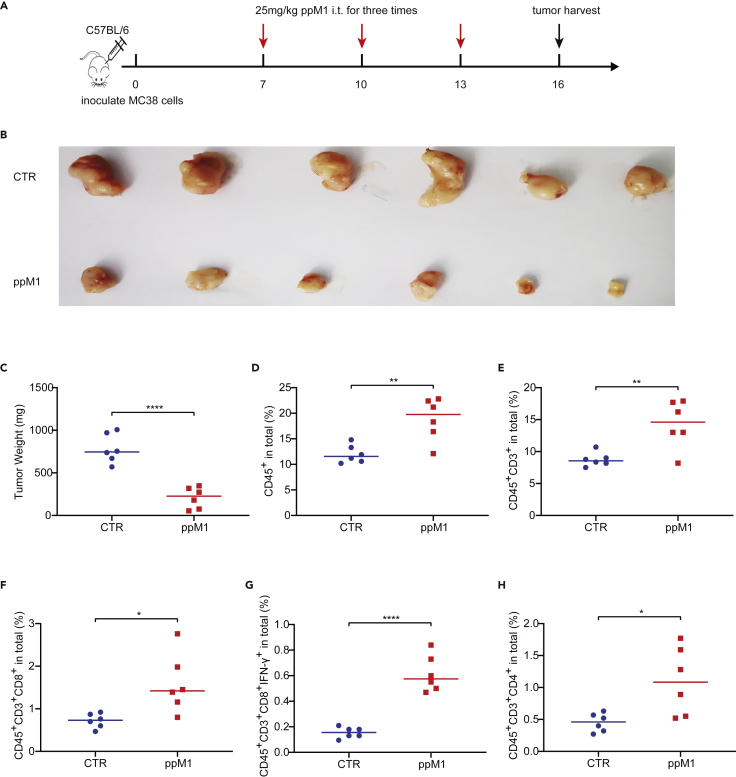
PpM1 treatment enhanced T cells infiltration and remolded the tumor immune microenvironment Tumors (n = 6) were harvested on day 16 and stained for an array of immune cell markers before being analyzed by flow cytometry. (A) The schedule of the experiment. (B and C) Tumor volume (B) and body weight of mice (C) on day 16 after tumor inoculation. (D–H) Immune profiling in MC38 tumor with or with ppM1 treatment, showing CD45^+^ leukocytes (D), CD3^+^ T lymphocytes (E), CD8^+^ T cells (F), IFN-γ^+^ cytotoxic T cells (G), and CD4^+^ T cells (H). Each dot represents data for one mouse and error bars represent SDs. (C–H) was analyzed with two-tailed unpaired t test; ∗p < 0.05; ∗∗p < 0.01; ∗∗∗p < 0.001; ∗∗∗∗p < 0.0001 (See also Figure S8).

ppM1-formed membrane nanopores induce ICD both *in vitro* and *in vivo*, which could remold tumor immune microenvironment (TIME) by recruiting immune cells. To test this supposition, we used two murine tumor models, MC38 model and 4T1 model, respectively. The mice bearing MC38 tumors or 4T1 tumors were intratumorally injected with 25 or 50 mg/kg of ppM1 for three times at an interval of 3 days (Figures 5A and S8A). On day 3 after the final injection, the tumors were harvested and analyzed for the infiltration of immune cells. Compared to the untreated mice, the tumors of the mice treated by ppM1 were significantly smaller (Figures 5B, 5C, and S8B). As shown in Figures 5D, 5E, S8C, and 8D, both CD45^+^ immune cells (indicated leukocytes) and CD3^+^ immune cells (indicated T lymphocytes) within the tumor were significantly increased by ppM1 treatment compared to the untreated tumors. Further analysis showed that ppM1 treatment significantly enhanced the infiltration of CD8^+^ T cells to the tumor (Figures S8F and S8E), especially in increasing the proportion of IFN-γ^+^ of CD8^+^ T cells (Figures S8G and S8F). Besides, an increased infiltration of CD4^+^ T cells was also observed in the ppM1-treated tumors (Figures S8H and S8G). Interestingly, in 4T1 tumors, ppM1 treatment selectively enhanced the expression of PD-1 on leukocytes (CD45^+^) (Figure S8H) but not the expression of PD-L1 on tumor cells (CD45^−^) (Figure S8I), suggesting that there would some benefits from ppM1 in combination with PD-1 antibody. Taken together, our results demonstrated that ppM1-generated ICD could recruit immune cells to infiltrate in TME, and remold the tumor immune microenvironment, finally, resulting in tumor suppression or tumor clearance.

### Durable antitumor effect of ppM1 treatment depended on T cells

**Figure 6:**
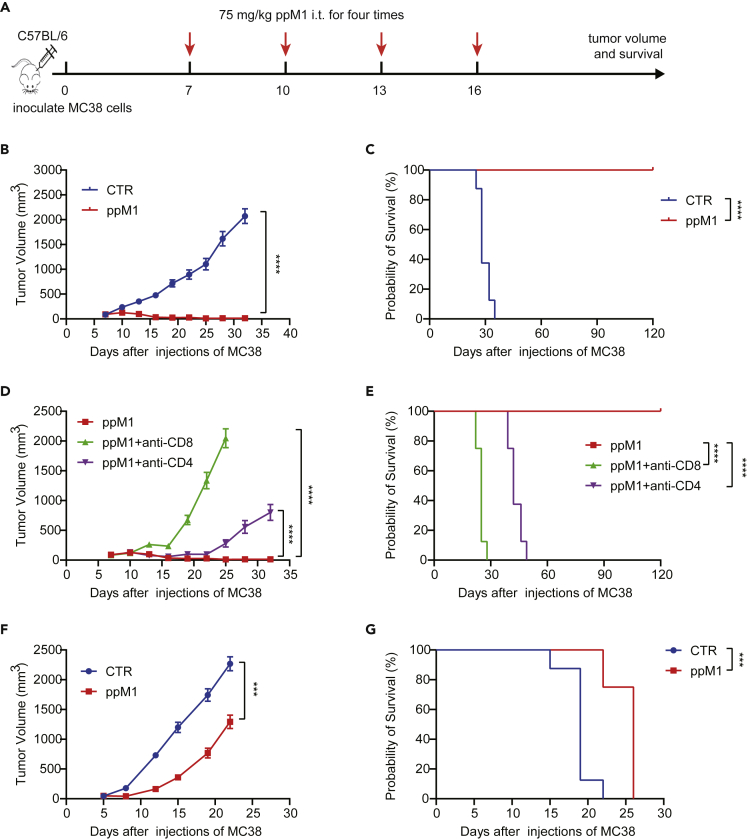
Durable antitumor effect of ppM1 treatment depended on T cells (A) The schedule of tumor therapy experiments in Figures 6B–6E, ppM1 treatment with or without CD8α antibody or CD4 antibody (200 μg/mouse) for three times for depleting CD8^+^ or CD4^+^ T cells on MC38-bearing C57BL/6 model (n = 6–8). (B and C) Tumor volume (B) and overall survival curves (C) of the groups without CD8 or CD4 depletion. (D and E) Tumor volume (D) and overall survival curves (E) of the groups with CD8 or CD4 depletion. (F and G) Antitumor efficacy of ppM1 on MC38-nude mice model (n = 8), tumor volume (F), survival curves (G). All error bars represent SEMs. (B), (D), and (F) was analyzed with two-tailed unpaired t test; (C), (E), and (G) was analyzed with log rank (Mantel–Cox) test. ∗p < 0.05; ∗∗p < 0.01; ∗∗∗p < 0.001; ∗∗∗∗p < 0.0001 (See also Figure S9).

To further evaluate the antitumor effect of ppM1, we raised the dosage of ppM1 to 75 mg/kg, and this dosage did not lead to the systemic toxicity to the mice (data no shown). The mice bearing MC38 tumor were intratumorally injected with ppM1 or vehicle for once every three days for four doses (Figure 6A). The dramatic antitumor effect was observed in the mice treated by ppM1, in which the curative rate was close to 100%. Five mice out of six were tumor-free, and the remaining one was with progression-free tumor, and all the mice were survival throughout the experiment period for 120 days. By contrast, the mice only received with vehicle were all dead within 35 days (Figures 6B and 6C). Next, we examined the importance of ppM1-generated ICD in its antitumor effect. Athymic Nu/Nu mice (which lack mature T cells) bearing MC38 tumors were intratumorally injected with ppM1, the tumor-free mice were not seen, only the delayed tumor growth and the limited extension of survival were observed in Figures 6F and 6G, respectively. Furthermore, following the procedure shown in Figure 6A, along with CD8 depletion or CD4 depletion, the durable antitumor effect of ppM1 vanished, no tumor-free, and just a retarded tumor growth could be seen (Figure 6D) with a little benefit for survival (Figure 6E). Taken together, these data revealed that ppM1 plays a dual effect of antitumor, including lymphocytes-independent antitumor, directly killing tumor cells by forming membrane pores, and lymphocytes-dependent antitumor, indirectly activating antitumor immune responses; both are interdependent to realize durable antitumor effect.

4T1 tumor, a murine triple-negative breast cancer, inherently resists to immunotherapy (Allen et al., 2020; Biswas and Mantovani, 2010). ppM1 treatment showed to remold the 4T1 TIEM and to specially increase the PD-1 expression on the leukocytes (Figure 5 and S8), indicating a benefit of the combination between ppM1 and immunotherapy is expectable, such as PD-1 antibody or MUC1 vaccine. The high expression of abnormal MUC1 in various human cancers (such as lung cancers and breast cancers) endows itself a rank of cancer-associated antigens (TAA)(Ho et al., 1993). Therefore, we transfected 4T1 cells with a vector encoding human MUC1 gene (4T1-MUC1), and then inoculated the cells to BALB/c mouse to evaluate the combination therapy of ppM1 and PD-1 antibody or MUC1 vaccine (Figure S9A). In line with the previous report, 4T1-MUC1 tumors also showed a resistance to PD-1 antibody or MUC1 vaccine alone treatment both in the tumor volumes (Figure S9D) and the overall survival of the treated mice (Figure S9E). Strikingly, 4T1-MUC1 tumors-bearing mice responded to ppM1 alone treatment and showed a significant decrease in tumor volumes (Figure S9B) and a prolonged survival (Figure S9C), compared to the untreated mice. As expected, compared to ppM1 alone treatment, both therapies of ppM1 in combination with either PD-1 antibody or MUC1 vaccine demonstrated further improved outcomes in the decreased tumor volumes (Figures S9F and S9H) and in the extended mouse life span (Figures S9G and S9I). The results give further evidence that ppM1 has a potential to remold TIEM, thus leading to enhancing tumor response to immunotherapy.

Moreover, B16F10 murine melanoma model was also employed to evaluate the therapeutic effect of ppM1 peptide *in vivo*. In addition, due to the metastatic property of B16F10, we also designed a metastatic tumor on the same mouse to evaluate the potent abscopal effects of ppM1 on distant metastases. On day 0, 2.5x10^5^ B16F10 melanoma cells were inoculated on the right flank of C57BL/6 mice as the primary tumor, and on day 7, 1x10^5^ B16F10 melanoma cells were inoculated on the left flank of mice as the metastatic tumor. From day 7, the ppM1 or saline was intratumorally injected into primary B16F10 tumor for three doses with a four days interval (Supplemental Figure S10A). Compared to the saline group, the dramatic antitumor effect was observed in the mice treated by ppM1 (Figure S10B). More importantly, 50% of mice (2 of 4) treated with saline alone developed distant metastases on the left flank by day 15, and all four mice developed distant metastases by day 22. However, by contrast, none of the ppM1-treated mice developed distant metastases (Figure S10C). These findings suggest that ppM1 is capable to induce immunogenic cell death in primary B16F10 tumor and exert optimal abscopal effects on distant metastatic tumor growth. The results provide experimental basis and theoretical basis for clinical translation.

## Discussion

**Figure 7:**
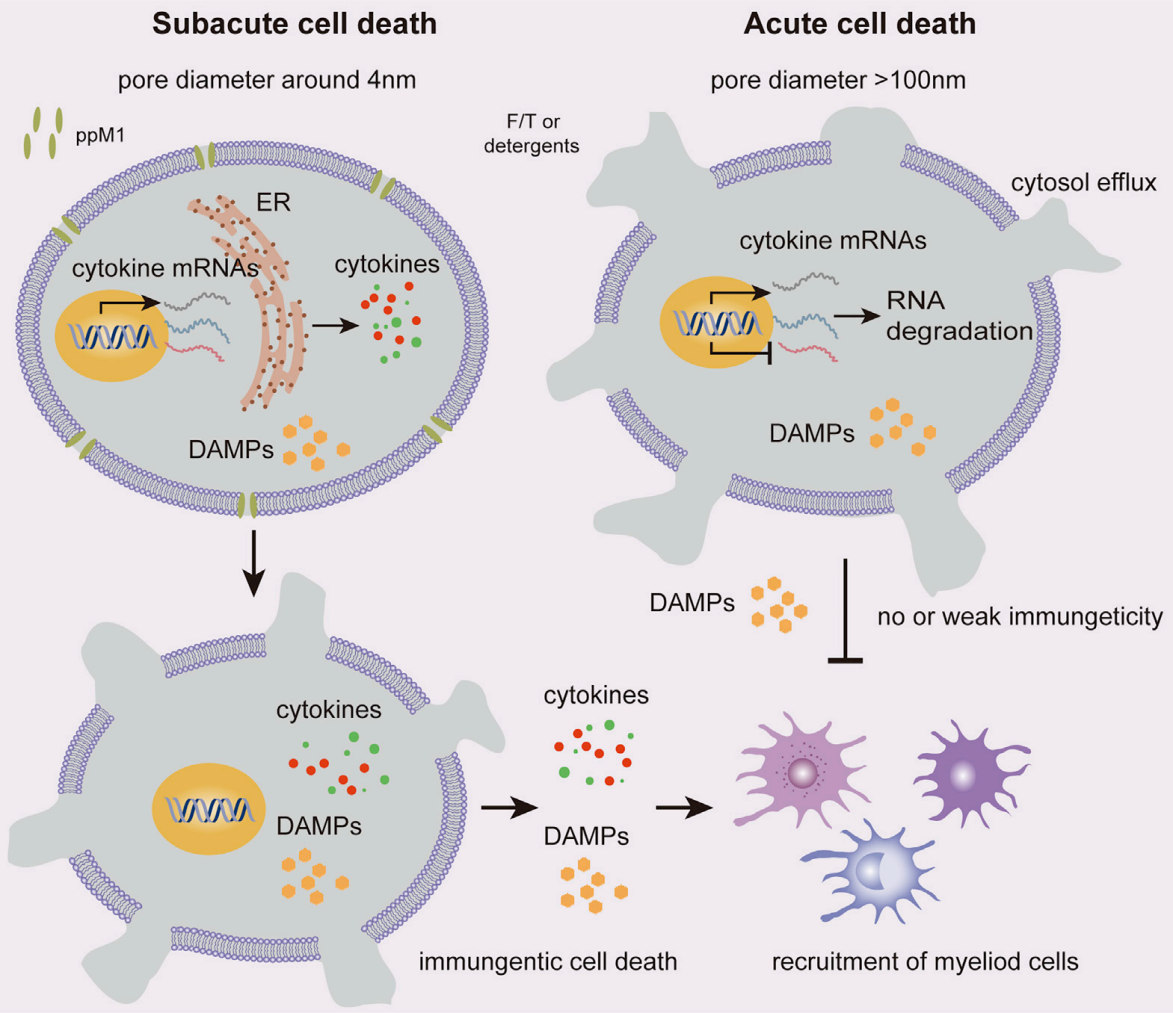
The mechanism scheme for poroptosis induced by ppM1 The nanopores formed by ppM1 was steady and irreversible and induced SCD of tumor cells. The cells being in SCD could maintain relative integrated subcellular organelle for a period of time, thus the genes transcription or proteins translation of cytokines could be continued, which would trigger a *bona fide* ICD, whereas acute cell death induced by F/T or detergents could not.

In this study, we reported a new peptide, ppM1, and revealed that ppM1 possesses the effect of oncolysis by forming nanopores on tumor cell membrane and then generates potential ICD to achieve the alteration of TIME, thus resulting in an antitumor immune response. The elimination of tumors by ppM1 treatment proceeded in three phases: ppM1 rapidly accumulated on tumor cell membrane to form nanopores and induced subacute cell death (SCD) (phase 1), which in turn resulting in cytokine and chemokine production that contributes to immune response to the dead cells (phase 2); the tumor cell undergoing ICDs that triggered transformation of TIME and activated an T cells-dependent antitumor immunity to clear remnants of tumor cells and prevent recurrence (phase 3). The first two phases could not only induce tumor cells into ICD but also reduce sharply tumor burden, offering better antitumor precondition for subsequent antigen-specific immune response or in combination with immunotherapy. The nanopores formed by ppM1 were steady and irreversible, which induced impaired membrane and fatefully deathward progress, that is SCD. The cells being in ppM1-induced SCD could maintain relative integrated subcellular organelle for a period of time, thus the genes transcription or proteins translation that associated with ICD could be continued. Plasma membrane nanopores-mediated relatively chronic cell death is dramatically district from F/T-induced quickly acute cell death (Figure 7). This process is characterized by forming the irreparable nanopore on plasma membrane and following by the sustained release of intracellular LDH. We named this irreparable plasma membrane nanopore-mediated immunogenic cell death as poroptosis, besides ppM1, this type of membrane nanopore caused by any other ways (Ros et al., 2017; Wang et al., 2020; Zhao et al., 2019).

We also endeavored to elucidate what plasma membrane pores could be associated with ICD, and proposed a peptide-based approach to induce ICD, by forming relatively steady transmembrane pores with a characteristic size (larger than 4 nm in diameter). In fact, the formation of transmembrane pores widely involves in multiple cell life activities in the natural world, such as cell death, protein translocation, pathogen infection, immunity, and defense. In mammalian immune system, immune cells use various membrane-pore-forming proteins (complement, perforin, perforin-2, granulysin, gasdermins, and MLKL) to induce microbe-infected host cells or tumor cells death, some of which trigger inflammation and sound alarms to recruit immune cells and activate a protective response, leading to control infection or tumorigenesis (Liu and Lieberman, 2020). These facts suggest some unknown relevance between membrane-pores-mediated death and immune activation. Here, we demonstrated that peptide-formed membrane pores could potentially induce tumor cells into ICD, and in addition, membrane pores formed by voltage or protein could also induce antitumor immune response as mentioned above, indicating that membrane-pores-mediated ICD (i.e., poroptosis) is independent on the way of pore formation, instead of the formed pore’s characteristics, such as size, stability, as well as durability. The transient transmembrane pores, such as mediated by low voltage, cell-penetrating peptide, usually fail to induce cell death due to the rapid repairment by the fluidity of biomembrane. Besides, eukaryotic cells respond to plasma membrane damage with a stereotypic membrane repair response, sometimes called cellular wound healing, that can rapidly restore membrane integrity. Those unstable transmembrane pores, mediated by mechanical stress or biochemical agents such as pore-forming toxins, could also been repaired rapidly in a variety of cellular models, including patching with endomembrane, endocytosis, and extracellular budding (Andrews and Corrotte, 2018; Cooper and McNeil, 2015; Jimenez et al., 2014; McNeil and Kirchhausen, 2005). Therefore, the most fundamental demand for inducing poroptosis of tumor cells was that the pores could be present steady and lastingly on plasma membrane, so that these pores can induce cells death no matter what response of membrane repair.

In the course of infection and immunity, pore-form-protein-induced abnormal cell death needs completely activated upstream and downstream signals to provide perfect precondition for subsequent immune response activation. Along with that, acute or accidental cell death, such as rapid disruption of plasma membrane, regardless of upstream signals is unlikely to induce ICD. In other words, the moribund cells need time to make a response to damage or danger and sound “alarm signals”; these alarm molecules could be cytokine and chemokine that determine the subsequent immune response activation or not; we call this kind of cell death as a subacute cell death (SCD) that is like-necroptotic cell death, an indispensable process of poroptosis. The SCD is a vital process for poroptosis, alternatively, the acute cell death induced by the oversize membrane damage is not; in this process, alarm molecular mRNAs are quickly degraded due to losing ER function, in which is essential for mRNA translation. The detail of mechanisms involving in poroptosis needs to be further investigated in the future. As shown in Figures 3E–3G and S4C–S4E, acute cell death caused by F/T-mediated membrane crack or detergents-mediated membrane lysis (that is oversize membrane pores) showed feeble immunogenicity to induce maturation of BMDCs, as a result that failed to generate antitumor immune protection (Figures 3I and 3J). Despite a correlation between several “danger signals” (such as ATP, HMGB1) and ICD has been reported (Menger et al., 2012), our work suggests that these markers are not enough to prediction and judgment of ICD; the process of death may be considered as a critical factor to induce ICD, involving the induction of cytokine and chemokine production. Thus, the membrane nanopores to induce ICD not only need the time to keep going long enough but also need the sizes to be confine within a certain range, that are too small to induce cell death by membrane repairing mechanism (smaller than 4 nm in diameter), while too large to synthetize cytokines that provoke host immune response (larger than 100 nm in diameter), these are also the essential requirements of poroptosis. In other words, inducing ICD of tumor cells must undergo the process of SCD, but SCD not always mean ICD because some tumor cells are with the innate deficiency of genes or proteins that associated with ICD. We believe that one way regardless of chemistry, physics, or biology, as long as it makes such transmembrane nanopores, could have a potential to induce tumor cell poroptosis.

Specific characteristics of plasma membrane nanopore in poroptosis are not completely clear. Poroptosis cannot be simply understood as a sustained release of intracellular LDH, because it induces immunogenic cell death. As the different mechanism of membrane pore formed by distinct approaches, there may be discrepant requirements in each approach-induced poroptosis. Our findings are most consistent with MLKL and GSDMA3 proteins-formed plasma membrane nanopores, respectively. The marked difference between them lies in an exogenous peptide and an endogenous protein. Membrane pore represents a type of plasma membrane injuries; selectively targeting cancer cell membrane to generate poroptosis could be a promise strategy for cancer therapy.

### Limitations of the study

Although, poroptosis has been characterized by the sustained release of LDH and the rate of cytokine mRNA degradation, which distinguish poroptosis from other forms of acute cell death. However, the above-mentioned characteristics are only based on indirect proofs, and the critical molecules and signal pathways that initiate or transduce this process remain to be elucidated by further explorations and efforts.

## STAR★Methods

### Key resources table

**Table:** 

REAGENT or RESOURCE	SOURCE	IDENTIFIER
**Antibody**		

InVivoMab anti-mouse PD-1 (CD279)	BioXCell	Cat # BE0273; RRID: AB_2687796
InVivoMab anti-mouse CD8a	BioXCell	Cat # BE0061; RRID: AB_1125541
InVivoMab anti-mouse CD4	BioXCell	Cat # BE0003; RRID: AB_1107638
InVivoMab rat IgG2a isotype control	BioXCell	Cat # BE0089; RRID: AB_1107769
Anti-mouse CD8a FITC	Biolegend	Cat # 100705; RRID: AB_312744
Anti-mouse CD8a APC-CY7	Biolegend	Cat # 100714; RRID: AB_312753
Anti-mouse CD4 PE	Biolegend	Cat # 100408; RRID: AB_312693
Anti-mouse CD274(PD-L1) PE (clone 10F.9G2)	eBioscience	Cat # 12-5982-81; RRID: AB_466088
Anti-mouse CD3 APC	Biolegend	Cat # 100236; RRID: AB_2561456
Anti-mouse CD3 PE-CY7	Biolegend	Cat # 100220; RRID: AB_1732057
Anti-mouse CD11c APC	Biolegend	Cat # 117310; RRID: AB_313779
Anti-mouse CD4 Brilliant Violet 421™	Biolegend	Cat # 100437; RRID: AB_10900241
Anti-mouse CD45 Brilliant Violet 605™	Biolegend	Cat # 103140; RRID: AB_2562342
Anti-mouse CD45 APC	Biolegend	Cat # 103112; RRID: AB_312977
Anti-mouse CD40 FITC	Biolegend	Cat # 124607; RRID: AB_1134090
Anti-mouse CD80 PE-Cy7	Biolegend	Cat # 104733; RRID: AB_2563112
Anti-mouse CD86 PE	Biolegend	Cat # 105007; RRID: AB_313150
Anti-mouse IFN-γ PE	Biolegend	Cat # 505808; RRID: AB_315402
Anti-mouse PD-1 FITC	Biolegend	Cat # 135213; RRID: AB_10689633
p53 (7F5) Rabbit mAb	Cell Signaling Technology	Cat # 2527; RRID: AB_10695803
MDM2 (D1V2Z) Rabbit mAb	Cell Signaling Technology	Cat # 86934; RRID: AB_2784534
β-Actin (D6A8) Rabbit mAb	Cell Signaling Technology	Cat # 8457; RRID: AB_10950489
Calreticulin Rabbit mAb (Alexa Fluor® 488 Conjugate)	Cell Signaling Technology	Cat # 62304; RRID: AB_2799626

**Chemicals, Peptides, and Recombinant Proteins**		

rmGM-CSF	Peprotech	Cat # 315-03
Collagenase IV	Invitrogen	Cat # 17104019-1
7-AAD Viability Staining Solution	eBioscience	Cat # 00-6993
Propidium Iodide Staining Solution	eBioscience	Cat # 00-6990
Thiazolyl Blue Tetrazolium Blue (MTT)	Sigma-Aldrich	Cat # M5655
Cholesterol	Avanti	Cat # 7000P
Dipalmitoyl phosphatidylcholine (DPPC)	Avanti	Cat # 850355
Glutaraldehyde	Sigma-Aldrich	Cat # G5882
Osmic acid	Avantor	Cat # 100504
Dodecyl sodium sulfate	Sigma-Aldrich	Cat # L3771

**Chemicals, Peptides, and Recombinant Proteins**		

Triton X-100	Sigma-Aldrich	Cat # T8787
PEG2000-PE	Avanti	Cat # 880120P
MPLA	Avanti	Cat # 699800P
Cholesterol	Avanti	Cat # 700000P
DPPC	Avanti	Cat # 850355C
PEG 4000	Sigma-Aldrich	Cat # 95904
PEG 8000	Sigma-Aldrich	Cat # 89510
MUC1 peptides (BLP25)	GuoPing Pharmaceutical	NA
pM1 and ppM1 peptides	GuoPing Pharmaceutical	NA
MitoTracker Deep Red	Thermo Fisher Scientific	Cat # M22426
Carboxy tetramethyl rhodamine	Thermo Fisher Scientific	Cat # 46112
Zeocin	Invivogen	Cat # ant-zn-05
RG-7112	Selleck	Cat # S7030

**Critical Commercial Assays**		

LDH Release Assay Kit	Beyotime Biotechnology	Cat # C0017
All-In-One MasterMix	Applied Biological Materials	Cat # 492
BCA Protein Assay Kit	Pierce	Cat # 23225
SYBR Select Master Mix	Applied Biosystems	Cat # 4472908
Fix/Permeabilization kit	eBioscience	Cat # 88-8823-88
10×RBC Lysis Buffer	eBioscience	Cat # 00-4300-54
Cell Lysis Buffer	Cell Signaling Technology	Cat # 9803
ATP Assay Kit	Beyotime Biotechnology	Cat # S0027
Mouse HMGB1 ELISA kit	Immunoway	Cat # KE1746

**Experimental Models: Cell lines**		

TC1 lines	ATCC	Cat # JHU-1
4T1 lines	ATCC	Cat # CRL-2539
4T1-MUC1 lines	This paper	
CT26 lines	Laboratory of Yangxin Fu	NA
MC38 lines	Laboratory of Yangxin Fu	N/A
MCF7 lines	ATCC	Cat # HTB22
A549 lines	ATCC	Cat # CRM-CCL-185
H1299 lines	ATCC	Cat # CRL-5803
SAOS-2 lines	ATCC	Cat # HTB-85
MRC5 lines	ATCC	Cat # CCL171
B16F10 lines	ATCC	Cat # CRL-6475
NIH/3T3 lines	ATCC	Cat # CRL-1658
MDA-MB-231 lines	ATCC	Cat # CRM-HTB-26

**Experimental Models: Organisms/Strains**		

C57BL/6	Vital River	Cat # VR21305
BABL/c	Vital River	Cat # VR21105
Nude	Vital River	Cat # VR40101

**Recombinant DNA**		

pcDNA3.1	Invitrogen	Cat # V79020
pcDNA3.1-Muc1	This Paper	

**Oligonucleotides**		

MDM2-F: GAATCATCGGACTCAGGTACATC	This paper	N/A
MDM2-R: TCTGTCTCACTAATTGCTCTCCT	This paper	N/A
Bax-F: CCCGAGAGGTCTTTTTCCGAG	This paper	N/A
Bax-R: CCAGCCCATGATGGTTCTGAT	This paper	N/A
Puma-F: GCCAGATTTGTGAGACAAGAGG	This paper	N/A
Puma-R: CAGGCACCTAATTGGGCTC	This paper	N/A
P21-F: TGTCCGTCAGAACCCATGC	This paper	N/A
P21-R: AAAGTCGAAGTTCCATCGCTC	This paper	N/A
GADD45b-F: TACGAGTCGGCCAAGTTGATG	This paper	N/A
GADD45b-R: GGATGAGCGTGAAGTGGATTT	This paper	N/A
IL-6-F: TAGTCCTTCCTACCCCAATTTCC	This paper	N/A
IL-6-R: TTGGTCCTTAGCCACTCCTTC	This paper	N/A
TNF-α-F: CAGGCGGTGCCTATGTCTC	This paper	N/A
TNF-α-R: CGATCACCCCGAAGTTCAGTAG	This paper	N/A
IFN-β-F: AGCTCCAAGAAAGGACGAACA	This paper	N/A
IFN-β-R: GCCCTGTAGGTGAGGTTGAT	This paper	N/A
CXCL1-F: CTGGGATTCACCTCAAGAACATC	This paper	N/A
CXCL1-R: CAGGGTCAAGGCAAGCCTC	This paper	N/A
CXCL2-F: TCCTCAGTGCTGCACTGGTC	This paper	N/A
CXCL2-R: CAGTTAGCCTTGCCTTTGTTCAG	This paper	N/A
CCL2-F: TTAAAAACCTGGATCGGAACCAA	This paper	N/A
CCL2-R: GCATTAGCTTCAGATTTACGGGT	This paper	N/A
CCL5-F: GCTGCTTTGCCTACCTCTCC	This paper	N/A
CCL5-R: CGAGTGACAAACACGACTGC	This paper	N/A
β-Actin-F: GTGACGTTGACATCCGTAAAGA	This paper	N/A
β-Actin-R: GCCGGACTCATCGTACTCC	This paper	N/A

**Software and Algorithms**		

GraphPad Prism 8	Graphpad Software	http://www.graphpad.com; RRID:SCR_002798
FlowJo	LLC	http://www.flowjo.com; RRID:SCR_008520
ImageJ		http://imagej.nih.gov/ig/; RRID:SCR_003073

### Resource availability

#### Lead contact

Further information and requests for resources and reagents should be directed to and will be fulfilled by the lead contact , Wei Liang (weixx@ibp.ac.cn).

#### Materials availability

Peptides generated in this study are available from the Lead Contact with a completed Materials Transfer Agreement.

### Experimental model and subject details

#### Cell lines

Murine breast cancer 4T1 and colon carcinoma CT26 (on BALB/c mice), cervical carcinoma TC-1 and colon carcinoma MC38, skin melanoma B16F10 (on C57BL/6 mice), human breast cancer MCF7 and MDA-MB-231, human alveolar basal epithelial cell cancer A549, human non-small cell lung cancer H1299, human osteosarcoma SAOS2, mouse embryonic fibroblast NIH/3T3, normal human fetal lung fibroblast cell MRC5 were cultured in 5% CO_2_ and maintained in RPMI 1640 or DMEM (McCoy’s 5A for SAOS2 cells) medium supplemented with 10% FBS (15% for SAOS2 cells), 100 U/ml penicillin, and 100 mg/ml streptomycin. The test for mycoplasma infection were negative.

#### Animals

Female C57BL/6, BALB/c or nude mice (6-8 weeks old) were purchased from Vital River Laboratory Animal Technology (Beijing, China). All animal experiments were performed according to the institutional ethical guidelines on animal care and the protocols used for this study were approved by the Animal Care and Use Committee at the Institute of Biophysics, Chinese Academy of Sciences.

### Method details

#### Cell viability assay based on thiazolyl blue tetrazolium blue (MTT)

Cells were seeded at a density of 0.5-1×10^4^ cells per well in 96-well plates and cultured for 24 h. Next day, cells were exposed to a series of concentrations of tested peptides for another 24 h, then the culture medium was discarded, and 100 μl MTT solution (with a working concentration at 0.5 mg/ml in PBS) was added to each well. After incubation at 37 °C for 4 h, the MTT solution was removed, and 200 μl of DMSO was added to each well for 10 min at room temperature. Absorbance was recorded at 590 nm by a plate reader (Thermo Multiskan MK3). The IC50s were calculated by nonlinear curve fit of log (inhibitor) vs. response on Graphpad prism8.0 software.

#### Cell viability assay based on propidium iodide staining

MC38 cells were seeded at a density of 2-5×10^5^ in 12-well or 24-well plates and cultured for 24 h. Next day, fresh medium with 5 μL of Propidium Iodide Staining Solution (per 1 ml medium) was replaced and cells were exposed to 30 μM of each tested peptide or PBS. After incubation at 37 °C for 30 min, cells were collected and washed once by FACS buffer or PBS solution, and then were analyzed on FACSCalibur (BD Biosciences) flow cytometer. The data were analyzed using FlowJo 10.0.8 software.

#### Stable cell line construction

The Muc1 gene was cloned from mouse lung tissue and inserted into pcDNA3.1 (Invitrogen) backbone plasmid. Muc1 expressing stable cell line 4T1-Muc1 was obtained by transfection with corresponding plasmid, selection in the presence of 100 μg/ml Zeocin (Invivogen), and cloning by limiting dilution.

#### LDH release assay

LDH release was performed as using LDH Release Assay Kit (Beyotime Biotechnology) as described in the instruction manual. Briefly, MC38 cells were seeded in plates. Next day, cells were treated with different reagents for a period of time as designed, then the medium were collected by centrifugation at 400g for 5 min at 4°C. LDH released into the cell medium supernatant was examined according to the manufacturer’s protocols. The amount of LDH release was calculated as follows: release (%) = (exp. value – blank control) / (high control- blank control) × 100.

The effect of PEGs of different sizes was assessed using LDH release assays. For PEGs blockade, MC38 cells were treated with 30μM ppM1 for 6 h at the presence or absence of PEGs (4000) or PEGs (8000) and LDH release was measured. We controlled that at this concentration of different PEGs were not toxic to the cells.

#### Hemolysis properties of peptides

The hemolytic activity of peptides was evaluated by measuring the amount of hemoglobin release in an assay using fresh mouse erythrocytes. Fresh blood was collected from C57BL/6 female mice and centrifuged at 750 g for 10 min at 4°C. Then, the supernatant was removed and the precipitated erythrocyte was washed with cold PBS for 3 times. The erythrocyte suspension was cultured with gradient concentrations of peptides (final concentration at 100, 50, 25, 12.5, 6.25 μM) at 37°C for 1 h, and then the cell suspension was centrifuged at 1000 g for 10 min and 100 μL of the supernatant was transferred into a 96-well plate. The absorbance of the supernatant was determined at 415 nm. PBS and 0.1% Triton X-100 served as negative and positive controls respectively. Hemolysis was assessed visually and calculated using the following equation: (%) hemolysis = (sample-negative control)/(positive control-negative control) ×100.

#### Fluorescence microscopy

##### For the confocal imaging of pM1-treated H1299

H1299 cells were seeded at a density of 1×10^5^ cells per dish in glass bottom petri dishes. Next day, the cells were incubated with 30 μM pM1 at 37 °C for 2 h, followed by 200 nM MitoTracker Deep Red loading for another 15 min to indicate vital mitochondria, or by DAPI at 1 μg/mL for 5-10 min to label nucleus. Images were acquired from three or more randomly chosen fields using a confocal microscope Olympus FV1000 (Tokyo, Japan). This experiment was performed in serum-free medium.

##### For the confocal imaging of ppM1-treated MC38

MC38 cells were seeded at a density of 1.5×10^5^ cells per dish in glass bottom petri dishes. Next day, the cells were incubated with 10 μM ppM1 in the presence or absence of 100 ng/ml carboxy-tetramethyl-rhodamine. Images were acquired from three or more randomly chosen fields using a confocal microscope Olympus FV1000 (Tokyo, Japan). This experiment was performed in serum-free medium.

#### RNA isolation and real-time quantitative PCR

A549 or MC38 cells were treated with peptides, detergents or freezing-thawing as designed. Then, mRNA was isolated and purified using TRIzol Reagent and was reverse transcribed using All-In-One MasterMix (Applied Biological Materials). Quantitative PCR was performed using SYBR Select Master Mix (Applied Biosystems) analyzed on QuantStudio 7 Flex (Applied Biosystems). Relative expression values were calculated using the ΔΔcycle threshold method.

#### Western blot analysis

A549 cells were treated with 30 μM pM1 for 24 h. Cells were collected and lysed in Cell Lysis Buffer containing 1 mM PMSF. The protein concentration for each sample was determined using BCA Protein Assay Kit (Thermo Fisher Scientific). The proteins were separated by SDS-PAGE and transferred to PVDF membranes. Western blotting was performed with anti-p53, anti-MDM2 and anti-β-actin antibodies (Cell Signaling Technology). After incubation with HRP conjugated secondary antibodies, blots were revealed using ECL western blotting substrate (Tanon).

#### Scanning electron microscopy and image processing

MC38 cells were plated on a round cover glass (diameter, 8 mm) at an appropriate density. Next day, cells were washed once and treated with 10 μM ppM1 for 10 min, fixed with 2.5% glutaraldehyde at 4 °C overnight followed by wash for three times, and get fixed with 1% osmic acid at room temperature for 1.5 h, and another wash for three times. After dehydrating in a graded ethanol-water series to 100% ethanol (20, 50, 70, 85, 90, 95, 100%), samples were put in the critical point dryer. Washing steps were performed with PBS, and 10 min for each wash. In critical point dryer, samples were flushed three times in CO2 in a graded series for 30 min. The temperature was raised to above 32 °C for 60 min. After the coating process, the samples are then mounted onto an SEM carrier and observed by cold field emission scanning electron microscope (Hitachi SU8010, Japan).

#### Fluorescein-loaded liposome and leakage assays

Fluorescein-loaded liposome was designed to detect membrane pore formed by ppM1. Liposome was prepared by film-rehydration method. Briefly, cholesterol and DPPC were dissolved in chloroform or methanol, the lipid mixture was cholesterol and DPPC in a molar ratio of 9:11. The solvent was evaporated under a stream of nitrogen, and the dry lipid film was rehydrated at 53 °C with 1-2 ml buffer (20 mM HEPES (pH 7.5) and 150 mM NaCl, 20 mg/ml carboxy-fluorescein or FITC-dextran). In order to obtain 100 nm size uniform liposome, the liposome dispersion was extruded through two sacks of 400, 200 and 100 nm pore size polycarbonate membrane (Whatman) using Mini-Extruder extrusion device (Avanti). And the liposome encapsulating fluorescein was separated from unencapsulated dye by repeated washing with buffer (20 mM HEPES (pH 7.5) and 150 mM NaCl) on a centrifugal filter device (Millipore).

The prepared liposome was cultured with gradient concentration of peptides (final concentration at 100, 50, 25, 12.5, 6.25 μM) at 37°C for 2h, PBS and 1% Triton X-100 served respectively as negative and positive controls. Centrifuging at 12000g for 20 min and 100 μL of the supernatant was transferred into a black 96-well plate. The fluorescence of the supernatant was determined by Varioskan Flash (Thermo). Leakage was assessed visually and calculated using the following equation: (%) leakage = (sample-negative control) / (positive control-negative control) ×100.

#### Analysis of DAMPs release

MC38 cells were seeded in 6- or 12-well plates, allowed to adhere overnight, and then treated with 30 μM ppM1 for 8 h (for HMGB1 release assays), 2 h (for ATP release assays) or 1 h (for detection of calreticulin exposure). Supernatants were collected by centrifugation for HMGB1 and extracellular ATP detection, and the cells were lysed for intracellular ATP detection. According to the manufacturer’s instructions, ATP quantification was performed by an enhanced ATP Assay Kit (Beyotime Biotechnology), HMGB1 was quantified by an ELISA kit (Immunoway). For detection of calreticulin exposure, cells were collected and washed once in PBS with 0.5% FBS, stained by Alexa Fluor® 488 Conjugate Calreticulin antibody as described in the instruction manual, washed once and 5 μL of 7-AAD staining solution was added to excluded the dead cells and then cells were analyzed on FACSCalibur (BD Biosciences) flow cytometer. The data were analyzed using FlowJo 10.0.8 software and the amount of calreticulin exposure to membrane was assessed by the mean fluorescence intensity of calreticulin antibody of live cells.

#### Analysis of BMDCs surface-marker expression

BMDCs were prepared from the femurs of C57BL/6 mice at 8-10 weeks of age and were cultured in RPMI 1640 medium with 10% FBS, 0.1% β-mercaptoethanol and 20 ng/ml rmGM-CSF for 7 days with two replenishments of medium without disturbing the cells. And prepared BMDCs cocultured with dead MC38 cells (at a ratio of BMDCs: MC38=1:10), which were subjected to induce respectively by 50 μM ppM1, three cycles of freezing-thawing (-80/37 °C), 80 μg/ml SDS or 0.1% Trion-x100. After 18 h co-culture, all the cells were collected and washed once in PBS with 0.5% FBS and stained by fluorescence-labeled antibody at 4 °C for 30 min. BMDCs were sorted by immunostaining using anti-CD11c antibody. Maturation of BMDCs was analyzed by anti-CD40, anti-CD80, anti-CD86 antibodies staining. All the samples were analyzed on the FACSCalibur (BD Biosciences) flow cytometer and the data were analyzed with FlowJo 10.0.8 software.

#### *In vivo* prophylactic tumor vaccination

*In vitro*, poroptosis was induced in MC38 or CT26 cells by incubating with 100 μM ppM1 for 6 h or accidental necrosis was induced by three times of freeze-thaw cycles (−80/37 °C). After induction, 3×10^6^ dead cells were s.c. inoculated on the left flank of mice (MC38 cells to C57BL/6, CT26 cells to BABL/C). On day 8 after vaccination, the mice were challenged subcutaneously on the opposite flank with 2.5×10^5^ homogeneous live cells. Tumor growth on the challenge site was recorded for up to 5 weeks after the challenge, and the shrinkage or absence of tumors were considered efficacious antitumor vaccination.

#### Tumor models

Tumor cells were injected into the 4th inguinal mammary fat pad at 2×10^4^ cells per mouse (in 4T1-Muc1 tumor-bearing BALB/c mice model), and subcutaneously injected at 4×10^5^ cells per mouse (in MC38 tumor-bearing C57BL/6 mice model), subcutaneously injected at 2.5×10^5^ cells per mouse on the right flank of mice as the primary tumor on day 0, and on day 7, subcutaneously injected at 1×10^5^ cells per mouse on the left flank of mice as the metastatic tumor (in B16F10 tumor-bearing C57BL/6 mice model), 2.5×10^6^ cells per mouse (in H1299 tumor-bearing nude mice model). Mice were randomized to treatment groups when tumors reached certain sizes. The CD8 or CD4 T cell depletion was performed by intraperitoneally injection of 200 μg anti-mouse CD8α or CD4 antibody on the day before ppM1 treatment. Tumor volumes were measured twice a week and calculated as length × width × width/2. The animals were euthanized when the tumor volume reached 2000 mm^3^.

#### Preparation of PEG-PE micelle-based vaccine

The PEG-PE micelle-based vaccine was prepared by film-rehydration method (ref. Cell Discovery, 2017). Briefly, PEG-PE was dissolved in chloroform. MPLA was dissolved in chloroform and methanol with a volume ratio of 2:1. MUC1 peptides (BLP25) were dissolved in methanol. Then, the components of mixture were PEG-PE, MPLA and peptides in a molar ratio of 180: 3:4. The organic solvents were removed using a rotary evaporator to form antigen peptide-containing lipid film. Then the lipid film was hydrated with sterile deionized H_2_O at 53 °C for 30 min under the protection of nitrogen.

#### Tumor tissue isolation and immune infiltration analysis assays

Tumor tissues were collected, minced into small pieces, and digested in 2 mg/ml collagenase Type IV at 37°C for 1 h. The digested tumor tissues were then filtered through a 70 μm cell strainer to make a single-cell suspension. Surface markers of cell samples were stained at 4°C for 30 min with antibodies: anti-CD45, anti-CD3, anti-CD4, anti-CD8α, anti-PD-1, anti-PD-L1. For intracellular staining, cells were fixed, permeabilized overnight at 4°C (Fixation/Permeabilization Concentrate and Diluent kit, eBioscience) and subsequently stained with anti-IFN-γ antibody. All the samples were analyzed on the FACSCalibur or FACSAria IIIu (BD Biosciences) flow cytometer and the data were analyzed with FlowJo 10.0.8 software.

### Quantification and statistical analysis

#### Statistical analysis

Statistical analysis was performed using Prism (GraphPad) Software. All p values were calculated by two-tailed unpaired or paired t test for two groups, oneway ANOVA plus Dunnett multiple comparisons for multiple groups, and log-rank (Mantel–Cox) test for survival analysis. A value of p < 0.05 was considered statistically significant (∗p < 0.05; ∗∗p < 0.01; ∗∗∗p < 0.001; ∗∗∗∗p < 0.0001).

## Data Availability

All data reported in this paper will be shared by the lead contact upon request. This paper does not report original code. Any additional information required to reanalyze the data reported in this paper is available from the lead contact upon request.
