# Supplementary material for: Poroptosis: A form of cell death depending on plasma membrane nanopores formation
Source: iScience. 2022 May 30;25(6):104481. doi: 10.1016/j.isci.2022.104481 (PMC9194171; doi:10.1016/j.isci.2022.104481)
Supplement: Document S1. Figures S1–S11 [file mmc1.pdf]

iScience, Volume *25*

Supplemental information

Poroptosis: A form of cell death depending on plasma membrane nanopores formation

Hao Li, Zihao Wang, Xiaocui Fang, Wenfeng Zeng, Yanlian Yang, Lingtao Jin, Xiuli Wei, Yan Qin, Chen Wang, and Wei Liang

Figure S1

A

pM1	RPPLSQDTFSRLWDLLPRYGRKKRRQRRR
pM2	DMTRVVDDCPRRDDCSRSRYGRKKRRQRRR
pM3	DMTRVVDDCPRRDDCSRRRYGRKKRRQRRR
pM4	DVRYLRRDNTFDDSVVVPYRPPRVGSRYGRKKRRQRRR
pM5	DVRYLRRDNTFDDSVVVPYRRRRRRRR
pM6	DGDRDFRMFDRLNRALRLDRYGRKKRRQRRR
pM7	SQIPASRQRTLGGDDLYRRDQQDIVYCSNRLLGR
pM8	RGAVTTSQIPASRQRTLVDPDYGRKKRRQRRR
pM9	DPDVLTRQRSAPIQSTTVAGRYGRKKRRQRRR
pM10	DRTYTMDRVLFYLGQYIMTDDYGRKKRRQRRR
pM11	DRTYTMDRVLFYLGQYIMTDDRRRRDRRD
pM12	RLLGRLFGVPSFSVKRDDDYGRKKRRQRRR
pM13	DRDDDIYTMIYDNLVVVNQQRYGRKKR RQRRR

B

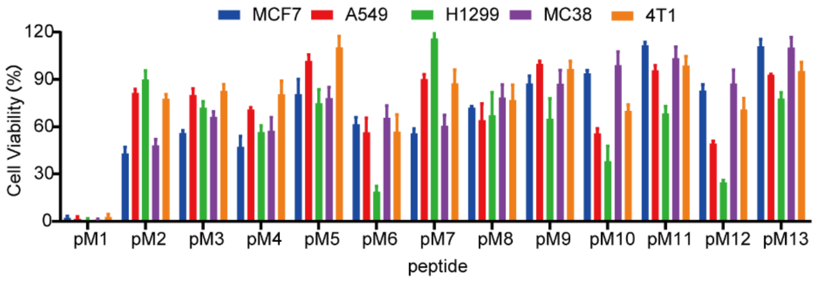

Figure S1. Tumoricidal effects of various peptides on several different tumor cell lines.

Related to Figure 1.

(A) Amino acid sequences of thirteen peptides.

(B) Cell viability of different tumor cells treated by 100 μM of various peptides for 24 h.

Data are representative of three independent experiments; all the error bars represent SDs.

Figure S2

A

pM1	RPPLSQDTFSRLWDLLPR-YGRKKRRQRRR
pM1-LG	RPPGSQDTFSRGWDGGPR-YGRKKRRQRRR
pM1-RG	GPPLSQDTFSGLWDLLPG-YGRKKRRQRRR
pM1-QG	RPPLSGDTFSRLWDLLPR-YGRKKRRQRRR
pM1-LA	RPPASQDTFSRAWDAAPR-YGRKKRRQRRR
pM1-LV	RPPVSQDTFSRVWDVVPR-YGRKKRRQRRR
pM1-LI	RPPISQDTFSRIWDIIPR-YGRKKRRQRRR

B

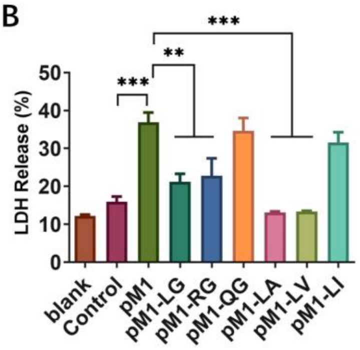

Figure S2. The charge of ppM1 peptide affects its toxicity to tumor cells. Related to Figure 1**.**

(A) pM1 polypeptides with different amino acid substitutions.

(B) LDH release of MDA-MB-231 tumor cells treated with pM1 polypeptides with different amino acid substitutions at a concentration of 100 μM

Figure S3

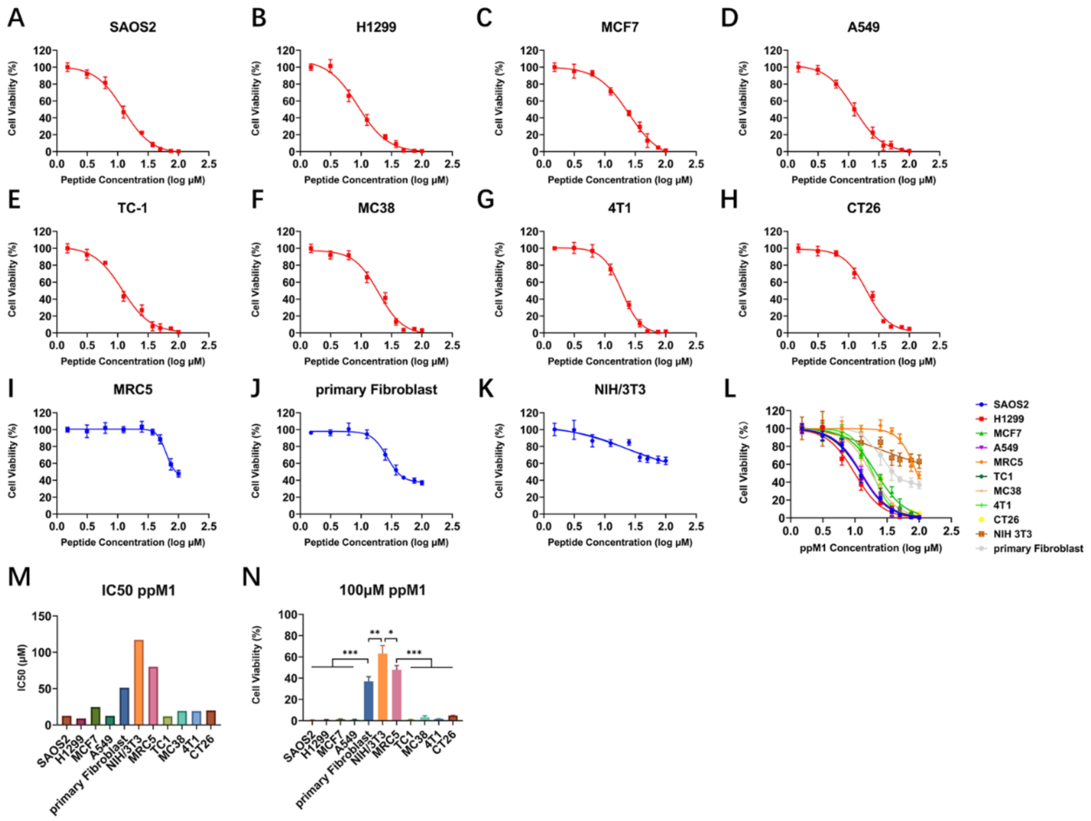

Figure S3. Cytotoxicity of ppM1 peptide on tumor cells and normal cells. Related to Figure 1.

(A-L) Survivorship curves of SAOS2, H1299, MCF7, A549, TC-1, MC38, 4T1, CT26, MRC5, primary fibroblast from mouse tail, NIH/3T3 cell lines treated with ppM1 for 24h.

(M) Comparison of IC_{50} between tumor cell lines and normal cells, measured by MTT experiments.

(N) Cytotoxicity evaluation by MTT experiment on several tumor cell lines and normal cells treated with 100 μM pM1 for 24 h.

Data are representative of three independent experiments; all the error bars represent SDs.

Figure S4

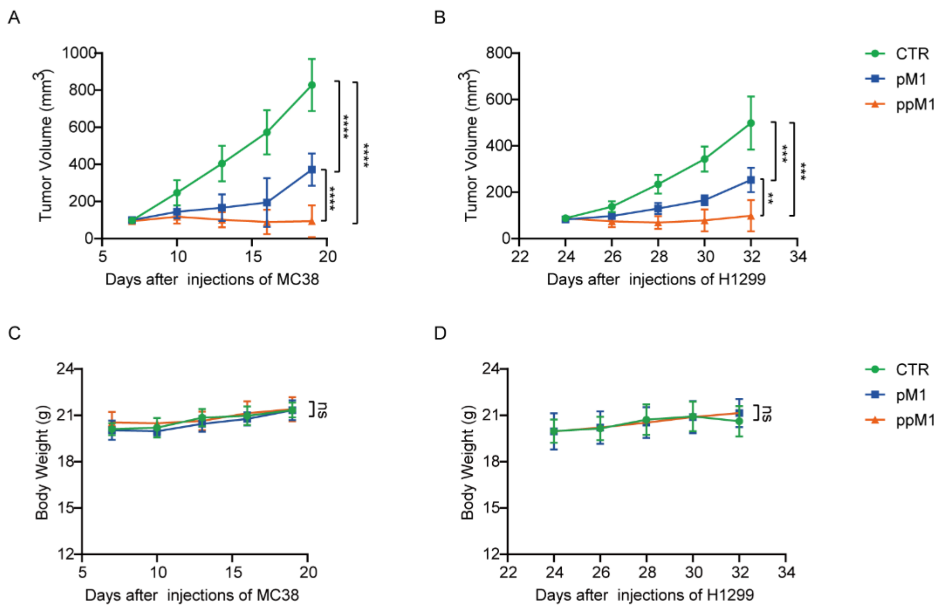

Figure S4. pM1 and ppM1 treatment suppressed tumor growth in vivo. Related to Figure 1.

Mice were treated with vehicle or pM1 or ppM1 after tumor volume reached 50-100 mm3 (intratumor injection 25mg/kg, every other day for 3 doses).

(A and C) Average tumor volume (A), average body weight (C) on MC38 tumor-bearing C57BL/6 mice (n=7-8).

(B and D) Average tumor volume (B) or average body weight (D) on H1299 tumor-bearing nude mice (n=6).

All error bars represent SDs. Data was analyzed with two-tailed unpaired t test. **p < 0.01; ***p < 0.001; ****p < 0.0001.

Figure S5

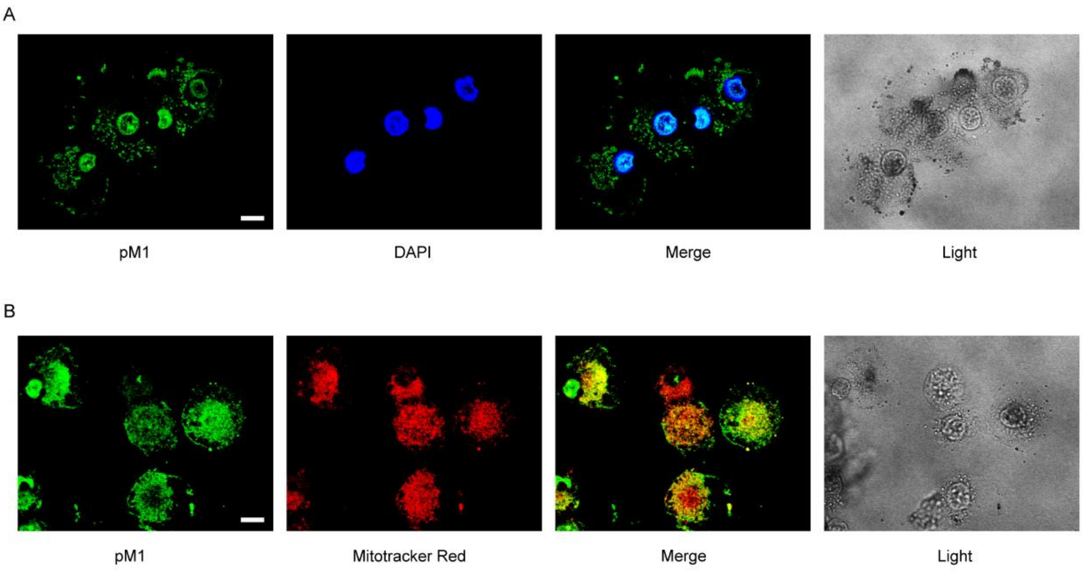

Figure S5. pM1 preferentially condensed in the nucleus after crossing plasma membrane. Related to Figure 2.

H1299 cell morphology observation after treated with 10 μM pM1 for 2h, green, blue and red was respectively representative of pM1-FITC, cell nucleus(A) and vitalized mitochondrion(B).

Figure S6

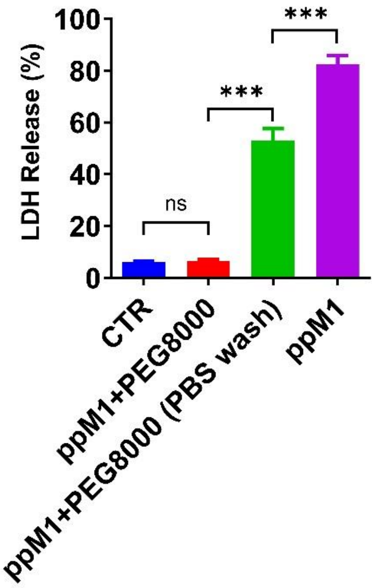

Figure S6. Removal of PEG8000 restored ppM1-induced MC38 cell death. Related to Figure 2.

Figure S7

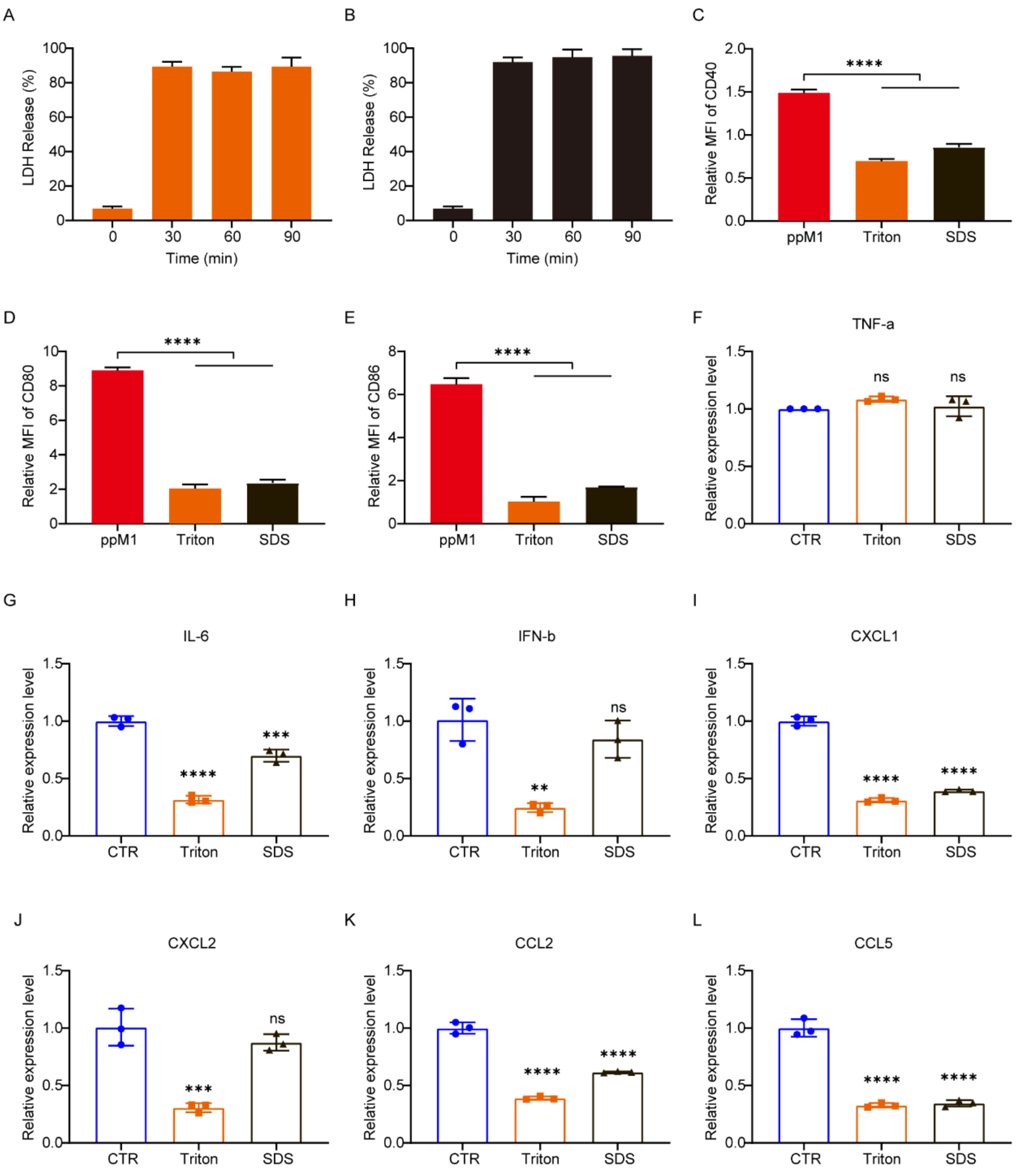

Figure S7. Detergents induced MC38 cell acute death and failed to result in ICD. Related to Figure 3 and Figure 4.

(A-B) Concentrations of extracellular LDH following MC38 cells treated by 0.1% Tritonx-100 (A) or 80 mg/ml SDS a(B) at various times.

(C-E) Flow-cytometry measurements of BMDC maturation markers, CD40(C), CD80(D), CD86(E) after coculturing with necroptotic MC38 cells induced respectively by ppM1, Tritonx-100 or SDS.

(F-L) Relative expression level of cytokine and chemokine genes following MC38 cells treated by 0.1% Tritonx-100 or 80 mg/ml SDS for 30 mins. TNF-a (F), IL-6 (G), IFN-b (H), CXCL1 (I), CXCL2 (J), CCL2 (K), CCL5 (L).

Data are representative of three independent experiments; all error bars represent SDs. (C-L) was analyzed with 1-way ANOVA. **p < 0.01; ***p < 0.001; ****p < 0.0001.

Figure S8

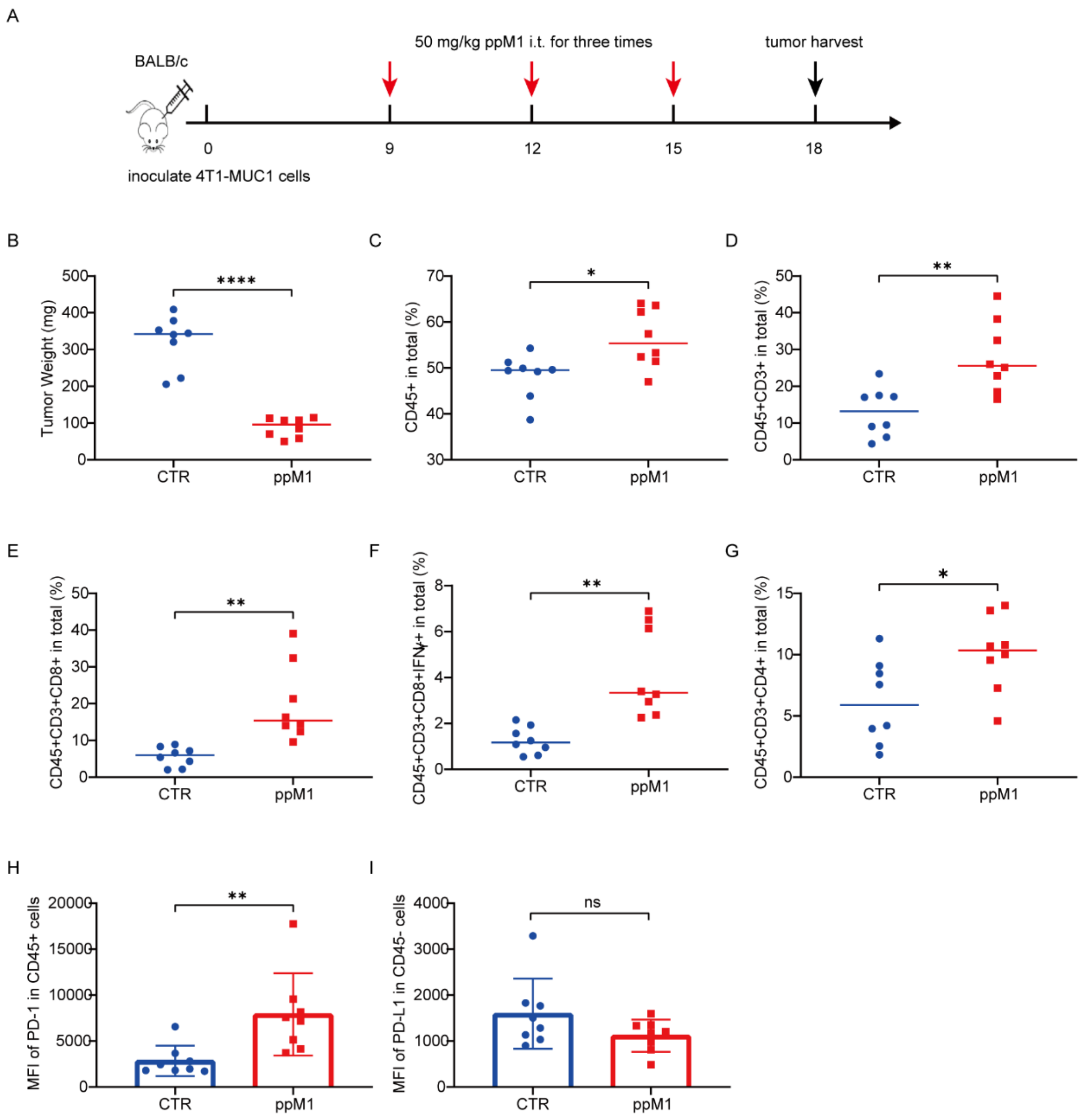

Figure S8. ppM1 treatment improved T cell infiltration on 4T1-MUC1 model. Related to Figure 5

4T1-MUC1 tumors (n=8) were harvested on day 18 and stained for an array of immune cell markers before analyzed by flow cytometry.

(A) The schedule of experiment.

(B) Weight of tumor harvested on day 18.

(C-I) Immune profiles assay of tumor with or without ppM1 treatment, CD45+ leukocytes (C), CD3+ T lymphocytes (D), CD8+ T cells (E), IFN-γ+ cytotoxic T cells (F), CD4+ T cells (G), mean fluorescence intensity of PD-1 on CD45+ cells (H), mean fluorescence intensity of PD-L1 on CD45- cells (I).

Each dot represents data for one mouse and error bars represent SDs in (B-I). Data was analyzed with two-tailed unpaired t test; *p < 0.05; **p < 0.01; ***p < 0.001; ****p < 0.0001.

Figure S9

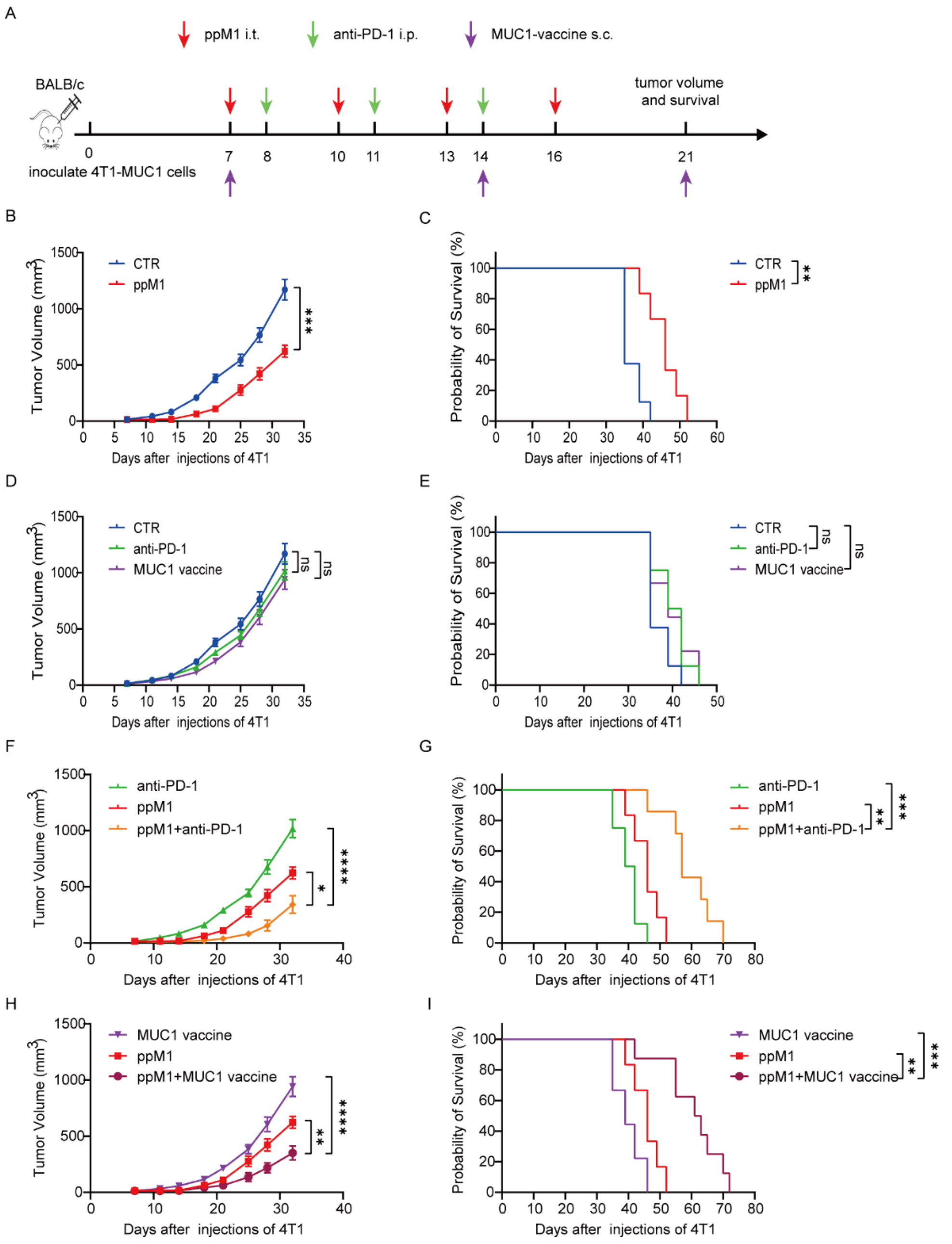

Figure S9. Antitumor efficacy of ppM1 or ppM1 combined with immunotherapy on 4T1-MUC1-BALB/c model. Related to Figure 5

(A) The schedule of experiment, ppM1(75mg/kg) was intratumorally injected (every 3 days, for 4 doses) from day 7 after tumor inoculation (n=6-9), PD-1 mAb (200 mg/dose/mouse) was i.p. injected (every 3 days, for 3 doses), MUC1 vaccine was s.c. injected (once a week, for 3 weeks). (B-I) Average tumor volume (B) (D) (F) (H), survival curves (C) (E) (G) (I).

All error bars represent SEMs. (B) (D) (F) (H) was analyzed with two-tailed unpaired t test; (C) (E) (G) (I) was analyzed with log-rank (Mantel–Cox) test. *p < 0.05; **p < 0.01; ***p < 0.001; ****p < 0.0001.

Figure S10

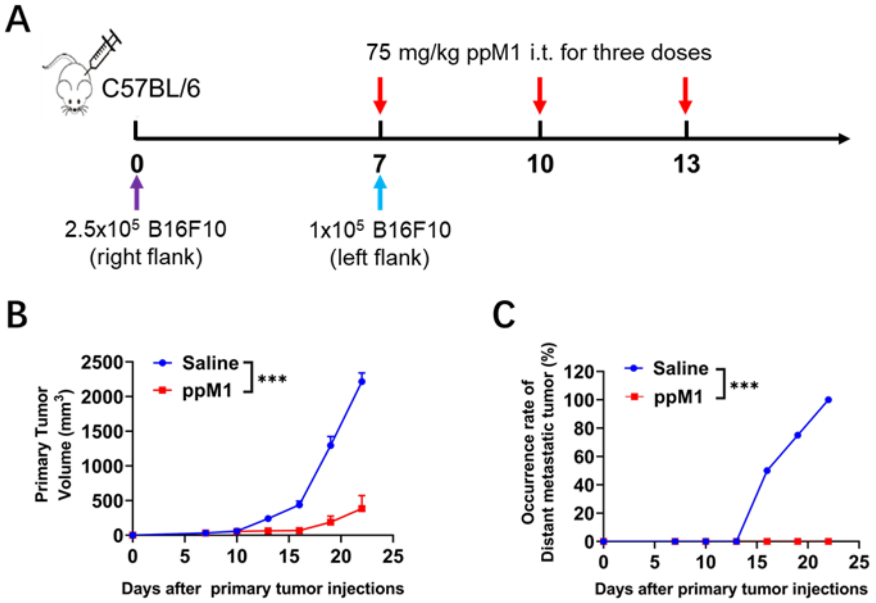

Figure 10. Antitumor efficacy of ppM1 on B16F10-C57BL/6 model. Related to Figure 6

(A) The schedule of experiment, ppM1(75mg/kg) was intratumorally injected (every 3 days, for 3 doses) from day 7 after primary tumor inoculation (n=4)

(B) Average primary tumor volume, (C) Occurrence rate of distant metastatic tumor

All error bars represent SEMs. (B) (C) was analyzed with two-tailed unpaired t test, ***$p < 0.001$;

Figure S11

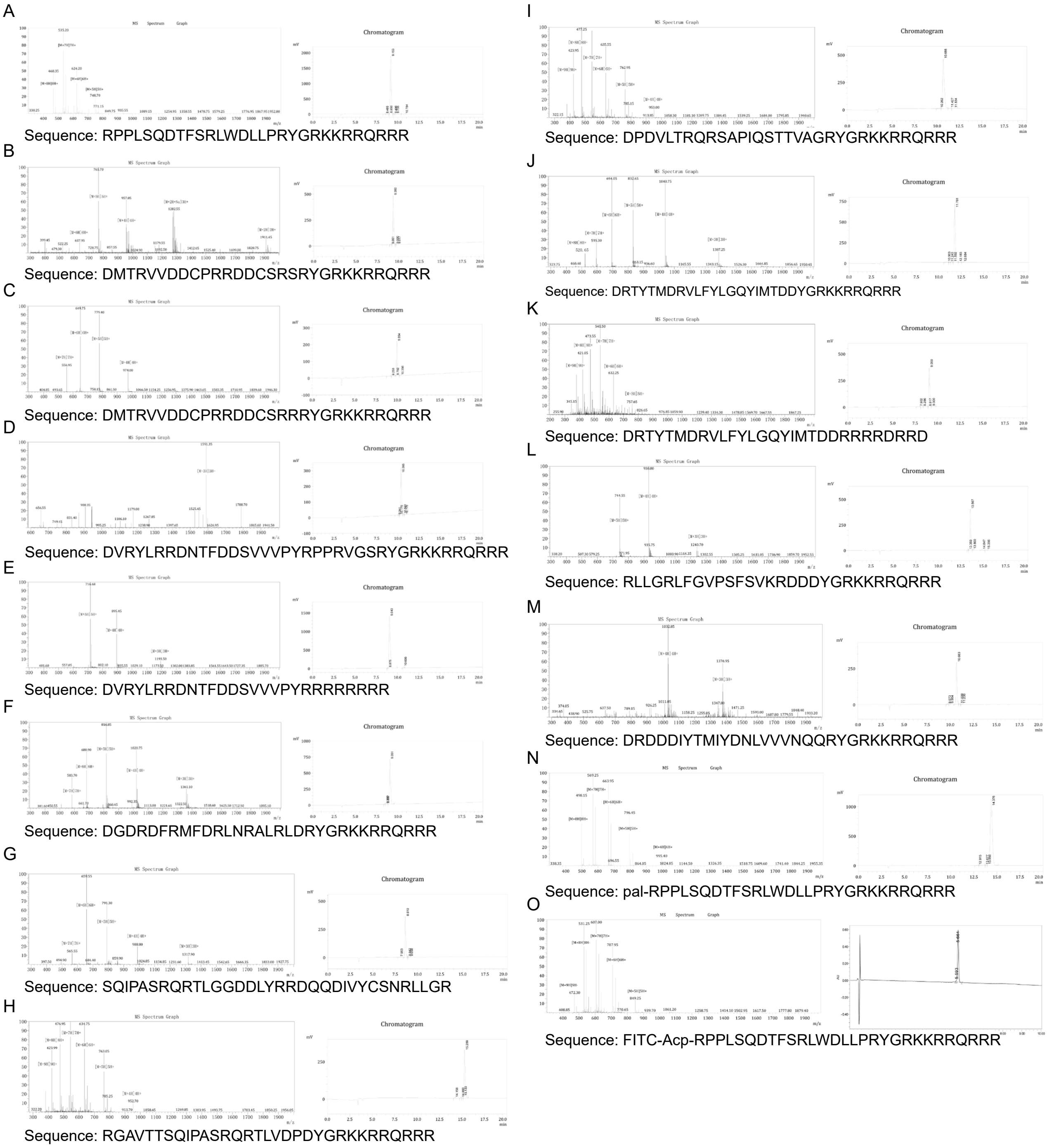

Figure S11. mass spectrometry and high-performance liquid chromatography results of the peptides in this study. Relate to Figure 1

(A-M) MS graph (left) and chromatogram (right) of pM1-pM13, respectively.

(N) MS graph (left) and chromatogram (right) of ppM1.

(O) MS graph (left) and chromatogram (right) of FITC-pM1.
